# Combining EEG and eye tracking: identification, characterization, and correction of eye movement artifacts in electroencephalographic data

**DOI:** 10.3389/fnhum.2012.00278

**Published:** 2012-10-09

**Authors:** Michael Plöchl, José P. Ossandón, Peter König

**Affiliations:** ^1^Institute of Cognitive Science, University of OsnabrückOsnabrück, Germany; ^2^Department of Neurophysiology and Pathophysiology, University Medical Center Hamburg-EppendorfHamburg, Germany

**Keywords:** eye tracking, EEG, independent component analysis (ICA), regression, artifact correction, eye movements

## Abstract

Eye movements introduce large artifacts to electroencephalographic recordings (EEG) and thus render data analysis difficult or even impossible. Trials contaminated by eye movement and blink artifacts have to be discarded, hence in standard EEG-paradigms subjects are required to fixate on the screen. To overcome this restriction, several correction methods including regression and blind source separation have been proposed. Yet, there is no automated standard procedure established. By simultaneously recording eye movements and 64-channel-EEG during a guided eye movement paradigm, we investigate and review the properties of eye movement artifacts, including corneo-retinal dipole changes, saccadic spike potentials and eyelid artifacts, and study their interrelations during different types of eye- and eyelid movements. In concordance with earlier studies our results confirm that these artifacts arise from different independent sources and that depending on electrode site, gaze direction, and choice of reference these sources contribute differently to the measured signal. We assess the respective implications for artifact correction methods and therefore compare the performance of two prominent approaches, namely linear regression and independent component analysis (ICA). We show and discuss that due to the independence of eye artifact sources, regression-based correction methods inevitably over- or under-correct individual artifact components, while ICA is in principle suited to address such mixtures of different types of artifacts. Finally, we propose an algorithm, which uses eye tracker information to objectively identify eye-artifact related ICA-components (ICs) in an automated manner. In the data presented here, the algorithm performed very similar to human experts when those were given both, the topographies of the ICs and their respective activations in a large amount of trials. Moreover it performed more reliable and almost twice as effective than human experts when those had to base their decision on IC topographies only. Furthermore, a receiver operating characteristic (ROC) analysis demonstrated an optimal balance of false positive and false negative at an area under curve (AUC) of more than 0.99. Removing the automatically detected ICs from the data resulted in removal or substantial suppression of ocular artifacts including microsaccadic spike potentials, while the relevant neural signal remained unaffected. In conclusion the present work aims at a better understanding of individual eye movement artifacts, their interrelations and the respective implications for eye artifact correction. Additionally, the proposed ICA-procedure provides a tool for optimized detection and correction of eye movement-related artifact components.

## Introduction

Neural activity measured with electroencephalography (EEG) or magnetoencephalography (MEG) yields a relatively weak signal, with amplitudes typically in the order of a few microvolts or femtotesla, respectively. At the same time, such measurements at scalp level are prone to electrical artifacts originating from non-neural sources such as the eyes, muscles or electrical devices in the surroundings. Compared to signals resulting from neural processes these artifacts can be several magnitudes larger in amplitude. Thus, cerebral activity may be buried in noise and remain undetectable even when a large number of trials is averaged.

A common strategy to circumvent this problem is to discard artifactual epochs from the data. This however often leads to considerable data loss, which in turn reduces the signal-to-noise ratio improvement that results from averaging techniques and therefore also the ability to detect neural activity. Additionally, this approach entails methodological constraints: in standard EEG or MEG experiments subjects are required to not move their eyes, thus adding unnatural cognitive loads to the experimental task and precluding the realization of EEG and MEG studies under more natural viewing conditions. Furthermore, the direct study of overt attention dynamics, by exploiting the temporal resolution of non-invasive electrophysiological methods, is rarely pursued. In studies that attempt to do so, the common practice is to analyze only the epochs before or after the eye movement and in this way to avoid problems arising from eye movement artifacts. However, some artifacts can extend to periods before and after saccade on- and offset, respectively, and thus the analysis of those periods may be confounded (Thickbroom and Mastaglia, 1985; Becker and Fuchs, 1988).

Likewise, studies that are not directly related to eye movements are also potentially impaired: when undetected, even small but systematic artifacts can add up over trials, thus distorting the analysis and the conclusions drawn from it (Hillyard and Galambos, 1970). In this context small involuntary eye movements during fixation, usually referred to as miniature eye movements (Rucci et al., 2007) or microsaccades (Martinez-Conde et al., 2004; Engbert, 2006), have recently received considerable attention, as they have been shown to vary systematically between different cognitive or perceptual states and in this way to introduce systematic biases in the analysis of neural activity (Yuval-Greenberg et al., 2008; Keren et al., 2010).

In order to overcome such limitations and biases, several procedures have been proposed in order to remove or at least reduce artifacts present in the data. These procedures include averaging, filtering (Luck, 2005), linear regression (Elbert et al., 1985; Croft and Barry, 2000a,b; Schlögl et al., 2007), Principal Component Analysis (Lins et al., 1993a; Kierkels et al., 2007), independent component analysis (ICA) (Jung et al., 2000; Iriarte et al., 2003; Joyce et al., 2004; Li et al., 2006; Mateenuddin et al., 2008), dipole modeling (Berg and Scherg, 1991; Lins et al., 1993a) and frequency methods (Croft and Barry, 2000a,b). Most of these approaches have been shown to effectively remove artifacts whose respective sources are well defined and whose spectral and statistical signal properties (e.g., amplitude, variance, frequency range, kurtosis etc.) differ considerably from those of neural activity. Averaging procedures for example substantially reduce random noise of moderate amplitude and therefore are standard in most electrophysiological experiments (Luck, 2005). Furthermore slow signal drifts caused by changing electrode properties, line noise and high frequency muscle activity can relatively easily be removed from the data by applying appropriate filters. Other muscle artifacts are reliably isolated into ICA components that can then be excluded from the data (Makeig et al., 1996; Jung et al., 2000; Iriarte et al., 2003). For eye movements on the other hand there is no standard correction procedure established yet, although several proposals do exist and are extensively discussed with respect to their efficiency (Croft and Barry, 2000a,b; Croft et al., 2005; Schlögl et al., 2007; Hoffmann and Falkenstein, 2008).

The difficulty to identify and correct eye movements can be largely attributed to the fact that a single eye movement produces several artifacts in the form of signal offsets and transients. These artifacts do not only emerge from different mechanisms but also differ in their statistical and spectral properties, depending on size and direction of the movement: while artifacts produced by eyeball rotation are the consequence of a direction change of the corneo-retinal dipole and therefore change roughly linearly with movement size and direction, blink artifacts are generated by the cornea being short-circuited to the extra ocular skin, thus being independent of corneo-retinal dipole orientation (Matsuo et al., 1975; Antervo et al., 1985; Chioran and Yee, 1991). In addition, the amplitude of the (pre-)saccadic spike potential, an artifact that most likely emerges from extra-ocular muscle activity, changes only marginally with saccade amplitude or direction (Keren et al., 2010; Carl et al., 2011). Therefore it may confound the data even during very small eye movements (i.e., microsaccades) that do not produce clearly visible corneo-retinal dipole offsets (Yuval-Greenberg et al., 2008). Another difficulty for the general characterization of eye movement artifacts is that there are also non-physiological factors that may alter the signal when eye movements are present in the data. As a result of high pass filtering during EEG recording, step-like signal changes, as they occur during eyeball rotation, cause the signal to slowly drift back toward its initial value according to the filter's cut-off frequency. Such drifts may confound the data up to several seconds after the actual saccade. Finally, re-referencing the data (e.g., to the average activity of all scalp electrodes) can change the sign and amplitude of a given artifact component at different recording sites, thus rendering its identification difficult.

In the literature different types of eye artifacts are usually reviewed individually (Thickbroom and Mastaglia, 1985; Chioran and Yee, 1991; Lins et al., 1993a,b; Keren et al., 2010). To our knowledge a comprehensive overview that also accounts for their interrelations during different saccade types does not exist.

Moreover, the heterogeneity of sources contributing to signal contamination by eye movements has important implications for correction procedures. Algorithms that assume that eye movement artifacts originate from a single source—or ignore that the relative contribution of different artifact sources to the signal may vary depending on the respective recording site and movement direction—will generally over- or under-correct the signal even if being accurate at one particular site. On the other hand, algorithms that can account for multiple and independent artifact sources (e.g., blind source separation methods) often depend on subjective decisions, as for example which of the isolated components relate to eye movements and therefore should be excluded from the data (Makeig et al., 1996; Jung et al., 2000).

In the following, using EEG and eye tracking during a guided eye movement paradigm, we will first review different types of artifacts produced by eye movements and investigate their projections to different electrode sites during a variety of saccades. Subsequently we will point out the advantages and disadvantages of different approaches to eye artifact correction and then propose a cleaning procedure to remove eye artifacts in an effective and objective manner.

## Methods

### Participants

We simultaneously recorded EEG and eye movements from 14 subjects (7 male, 7 female; age range: 20–31 years) after they were informed about the procedure and purpose of the study and had signed an informed consent. Experimental procedures conformed to the Declaration of Helsinski and national guidelines. All subjects were students at the University of Osnabrück and received payment or course credits for their participation. All subjects reported normal or corrected-to-normal vision.

### Stimulus presentation

The participants sat at 60 cm distance from a 30″ TFT monitor (Apple LED Cinema Display, refresh rate 60 Hz, resolution 2560 × 1600 pixels). Due to the range of the eye tracker only a square region of 960 × 960 pixels (~23° × 23°) in the center of the screen was used for stimulus presentation. The stimulus itself constantly covered about 3° of the visual field and consisted of black and white rings (see Figure 3), which continuously contracted toward the center at a rate of 2 Hz (i.e., when the outer ring started contracting into the center it was replaced by the next ring of the other color).

### Experimental procedure

Previous to each block of the actual experiment, subjects performed a short pre-experimental procedure in order to calculate the regression coefficients (Schlögl et al., 2007) and to complement the data for ICA decomposition (see below). This pre-experiment consisted of 16 trials of 15 s duration in which subjects were asked to perform different eye movements over a gray screen (RGB 127/127/127). Every subject performed four trials for each of the following movements: blinks, vertical movements, horizontal movements, and blinks plus vertical movements. On average each subject performed 169 blinks, 561 vertical, and 312 horizontal saccades during the pre-experiment.

**Figure 1:**
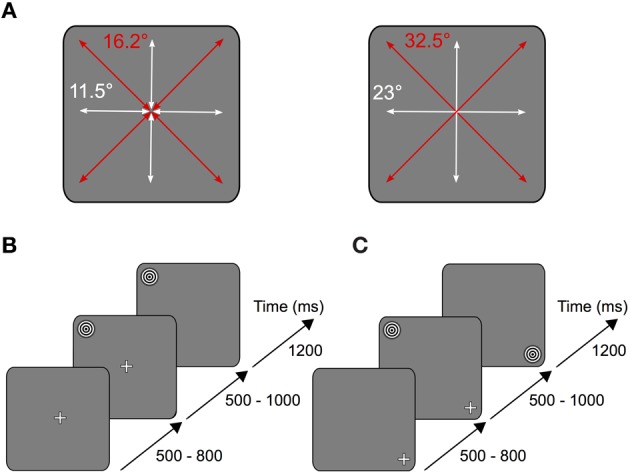
**Experimental task. (A)** Depending on the stimulus location relative to the fixation cross, subjects performed horizontal and vertical (white arrows) or oblique (red arrows) saccades on the screen. Left: short saccades (11.5° vertical and horizontal, 16.2° oblique) were either performed from the periphery to the center or from the center to the periphery. Right: long saccades (23° vertical and horizontal, 32.5° oblique) were performed from the periphery to another peripheral point located opposite across the center. **(B)** Each trial started as soon as the subject began to fixate the fixation cross located in one of nine possible locations on the screen. After a variable time of 500–800 ms the stimulus was shown in one of the other eight remaining locations on the screen. In the saccade condition the fixation cross disappeared after another 500–1000 ms, thus serving as the cue for the subject to make a saccade onto the stimulus. **(C)** In the fixation condition, instead of the fixation cross disappearing, the stimulus was relocated onto the position where the subject fixated.

After the pre-experiment the participants performed the task illustrated in Figure 1: a white fixation cross (size ~1.5°) appeared on a gray screen (RGB 127/127/127) in one of nine possible locations arranged in a three by three square. The respective trial started as soon as the subject started fixating the fixation cross. After a variable time (500–800 ms) the stimulus was presented in one of the other eight remaining positions on the screen (Figure 1A).

In one condition (saccade condition) the fixation cross disappeared after another 500–1000 ms, thus providing the cue for the subject to make a saccade to the stimulus location (Figure 1B). In the other condition (fixation condition), instead of the fixation cross disappearing, the stimulus disappeared in its original location and replaced the fixation cross on the fixated position (Figure 1C). Thus, at each location stimulus foveation could result from either an eye movement or the sudden appearance of the stimulus. The stimulus was presented for 1200 ms after disappearance of the fixation cross (saccade condition) or relocation of the stimulus (fixation condition), respectively. Each experimental block consisted of 480 saccade trials and 480 fixation trials, which were randomly interleaved. All subjects performed at least two pre-experimental and two experimental blocks.

### Eye tracking

Eye movements were recorded with a remote video eye tracking system using monocular pupil tracking at 500 Hz (Eyelink 1000, SR Research Ltd., Mississauga, Canada). To calibrate eye position a 13-point grid was used and the calibration procedure was repeated until the average error was below 0.5°. Saccades were defined using a velocity threshold of 30°/s, an acceleration threshold of 8000°/s^2^, and a minimum deflection threshold of 0.1°.

### Microsaccade detection

Using an algorithm published by Engbert and Mergenthaler (Engbert and Mergenthaler, 2006), microsaccades were defined as intervals in which the recorded eye movements exceeded a relative velocity threshold of six median-based standard deviations for a duration of at least six samples (12 ms) and had an amplitude between 0.1° and 1°. Microsaccade detection was only performed on fixation trials that did not contain any saccades larger than 1° (according to the criteria described in “Eye Tracking”).

### EEG recordings

EEG-data were recorded using an ActiCap 64-channel active electrode system with a BrainAmp DC amplifier (Brain Products GmbH, Gilching, Germany). 61 of the electrodes were placed equidistantly on the scalp, one was placed on the forehead (approximately 25 mm above the nasion), and another two on the left and right infraorbital rim, respectively. The impedance of all electrodes was reduced below 5 kΩ. The data were recorded at a sampling rate of 1000 Hz and online band-pass filtered between 0.016 Hz and 250 Hz. During recording all electrodes were referenced to a nose tip electrode.

### Preprocessing and analysis

All data were preprocessed and analyzed in Matlab (Mathworks) and using FieldTrip (Oostenveld et al., 2011) and EEGLAB (Delorme and Makeig, 2004).

EEG data were down-sampled to 500 Hz in order to match the sampling rate of the eye data and low-pass filtered at 100 Hz. Eye- and EEG data were then aligned by cutting them into trials according to the triggers that were simultaneously sent to both, the EEG and the eye tracking system. We visually inspected the EEG data and removed trials containing high amplitude noise, as it typically arises from muscle activity related to larger body movements, as well as blinks and other easily identifiable confounds such as sudden electrode drifts and jumps. Trials containing saccade-related artifacts were not excluded from analysis unless (1) they were not related to the task and exceeded 2° of visual angle in the saccade condition or 1° during fixation trials, (2) there was no stable fixation within 400 ms after the cue, or (3) they were not located in a window of 2° around the center of the stimulus.

Where explicitly stated in the results section, the EEG data were re-referenced to the average activity of the 61 scalp electrodes.

### Event-related potentials

For the analysis of event-related potentials (ERPs) the trials were cut into 1 s long epochs ranging from −500 to +500 ms around the event of interest (i.e., saccade on- or offset in the saccade condition and stimulus relocation in the fixation condition). Subsequently the trials were baseline corrected to the pre-event interval and then averaged for each condition over all subjects. Deviations from this procedure will be detailed in the respective result sections.

### Time-frequency analysis

Time-frequency analysis of the gamma frequency band (>30 Hz) was performed using a set of complex Morlet wavelets with a width of five cycles per frequency. The spectral estimate was obtained for frequencies ranging from 30 to 100 Hz in steps of 2 Hz.

For the analysis of the frequency bands below 30 Hz we used Fast Fourier Transform instead of wavelet analysis in order to obtain a constant frequency resolution. More specifically we segmented each trial into 200 ms long overlapping data windows, advancing in 5 ms steps from −500 to +500 ms. We then multiplied each data segment with a Hanning window before the Fast Fourier Transform was computed.

Finally, in order to obtain power changes with respect to baseline, all time-frequency bins were normalized to the average pre-stimulus power in each frequency bin and then averaged over trials and subjects.

### Independent component analysis

**Figure 2:**
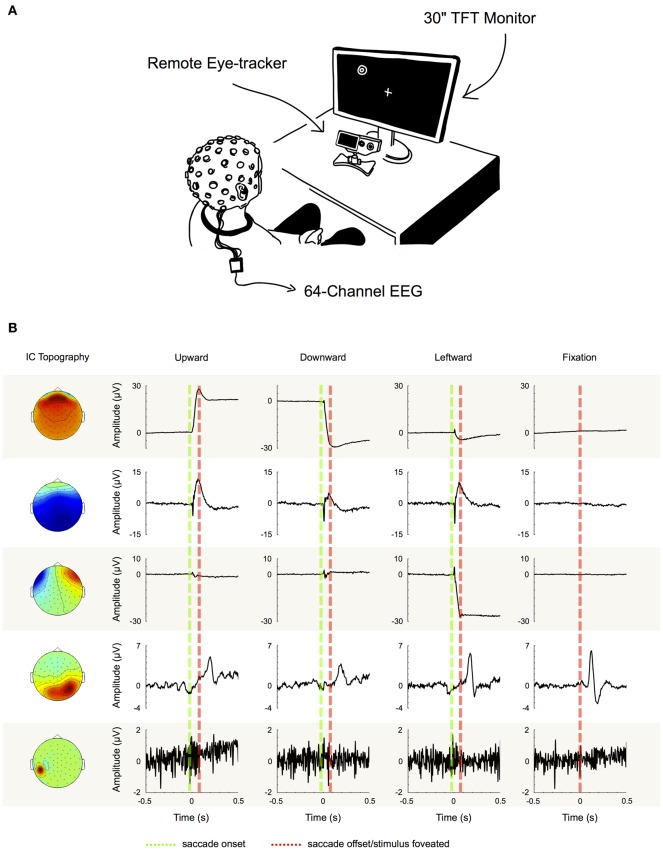
**Component examples and correction procedure. (A)** Experimental setup. **(B)** Examples of five typical IC topographies and their activations, as we found them similarly for all of our subjects. The first three ICs can be classified as eye movement-related as their activations display high variance during saccade intervals (between green and red dotted line), while being inactive during fixation periods (left and right of green and red dotted line). IC 4 on the other hand displays its largest variance during fixation. Therefore, it cannot be attributed to artifacts produced during saccade execution, but instead to neural activity—in this case the time locked visual response to the stimulus. Finally, the topography and signal properties of IC 5 suggest that it emerges from muscle activity or other noise sources at one particular electrode site. The component's activity is not systematically related to saccade execution or stimulus presentation and thus displays similar variance during both, saccade and fixation intervals. Based on these observations we used the variance difference between saccade and fixation periods in each IC to objectively differentiate eye artifact-related ICs from those related to neural activity and other sources.

We performed ICA to identify and extract ocular artifact components from the data. ICA is a blind source decomposition algorithm that enables the separation of statistically independent sources from multichannel data. It has been proposed as an effective method for separating ocular movement and blink artifacts from EEG data (Jung et al., 2000; Iriarte et al., 2003; Hoffmann and Falkenstein, 2008). ICA was performed using the Infomax ICA algorithm (Bell and Sejnowski, 1995) as implemented in the EEGLAB toolbox (Delorme and Makeig, 2004). In order to optimize the ICA decomposition with respect to eye movement- and blink-related components, we appended the experimental data with data from a pre-experimental procedure during which each participant performed blinks and vertical and horizontal saccades within the same region of the gray screen that would later be used for stimulus presentation (see experimental procedure). Then, for each subject individually, we decomposed the preprocessed data from all 64 channels into 64 statistically independent components (ICs). To differentiate ICs that are related to eye movements and blinks from the ones produced by neural activity and other sources, we followed the procedure that is illustrated in Figure 2B: first we partitioned every trial into saccade and fixation epochs. Saccade epochs were defined as the time between saccade on- (Figure 2B, green dotted lines) and offset (Figure 2B, red dotted lines) as given by the eye tracker. To ensure that both, spike potentials and post-saccadic eyelid artifacts were comprised by saccade epochs as well, we additionally included the intervals 5 ms before and 10 ms after saccade on- and offset, respectively. Conversely, fixation intervals were defined as the time between saccade epochs. Subsequently, for each trial, we computed the variance of the respective IC activations during saccades and during fixations (note that in Figure 2 for illustration purposes we show IC ERPs instead of IC activations during single trials). If for a given IC the mean variance of saccade epochs was at least 10% higher than the mean variance of fixation epochs (i.e., variance_saccade_/variance_fixation_ > 1.1), the IC was classified as eye-artifact-related and subsequently removed from the data. The threshold of 10% was introduced to avoid that components with constant variance over both, saccade and fixation epochs, might be misclassified due to random fluctuations.

Note that here we only rejected eye-artifact-related ICs, while ICs related to non-ocular artifacts (e.g., muscle activity) were not excluded from the data.

### Regression

We used two different linear models for regression-based artifact correction. In both models electrooculogram (EOG) channels were used as the independent variables that convey the signal of a single artifact source for both eyes. As the movement of the eyeball with respect to the head can be explained with only two spatial components, the first model only takes vertical and horizontal EOG measurements into account:

$$\text{EEG}{\left(t\right)}_{\text{observed}}=\text{EEG}{\left(t\right)}_{\text{source}}+\beta_{1}\text{vEOG}(t)+\beta_{2}\text{hEOG}(t).$$

It has been proposed that artifacts related to eyelid movement, which are independent of eyeball movement could be modeled as the third spatial component (radial) of the same single source that explains artifacts produced by eye movements (Elbert et al., 1985; Croft and Barry, 2000a,b; Schlögl et al., 2007):

$$\text{EEG}{\left(t\right)}_{\text{observed}}=\text{EEG}{\left(t\right)}_{\text{source}}+\beta_{1}\text{vEOG}(t)+\beta_{2}\text{hEOG}(t)+\beta_{3}\text{rEOG}(t).$$

For the model based on two spatial components, horizontal and vertical EOG channels were obtained using a triangular montage, consisting of one electrode on the forehead (e1) and two on the left (e2), and right (e3) infraorbital rim (cf. Schlögl et al., 2007). For the three spatial components model, besides the triangular montage a left (e4) and a right (e5) temporal electrode were included to generate the radial component (Elbert et al., 1985) according to e1/2 + (e2 + e3)/4 − [(e4 + e5)/2].

The coefficients for both models were obtained from the eye(lid) movements in the pre-experimental periods described above. The subjects' individual coefficients were then applied to the data of the experimental conditions in order to correct eye movement-related artifacts.

### Statistics

To test for statistical significance and at the same time to control for multiple comparisons, we used a cluster-based non-parametric permutation test that is described in detail in Maris and Oostenveld (2007). The rationale behind this test is summarized as follows: if for example the difference between two ERPs yields 20 samples that reach significance (e.g., according to a *t*-test), they are more likely to represent a difference in neural processing when they occur adjacent in time, as compared to when they occur at 20 independent time points (which rather would suggest random fluctuations in the signal). If at the same time significant values are also observed in several neighboring electrodes, the likeliness of these values being the result of neural activity is even higher. Following this rationale, ERP differences or time-frequency differences that exceed a predefined threshold (here two standard deviations from the mean) are clustered and summed across adjacent channels, time points and, for time-frequency analysis, across frequency bins. By randomly exchanging conditions in a random subset of subjects, i.e., flipping the sign of the observed values in both conditions before averaging and clustering, an alternative observation is obtained. Repeating this procedure multiple times (*n* = 1000) yields a reference distribution under the null hypothesis. It now can be tested how often clusters of the observed size are expected to randomly occur under the null hypothesis. Additionally, for statistical comparisons between multiple factors we used repeated-measures ANOVA.

## Results

### Stimulus response

We aim at investigating the impact of different types of eye movement artifacts on EEG data and evaluate the efficiency of regression and ICA-based artifact correction methods. As a reference for distinguishing neural activity from confounds induced by ocular artifacts we first assessed EEG responses to a standard visual stimulus during fixation. The stimulus of choice consisted of black and white contracting rings, because they are known to generate both, a clear visual ERP and a distinct gamma band response in human EEG and MEG signals (Hoogenboom et al., 2006; Fries et al., 2008; Koch et al., 2009).

**Figure 3:**
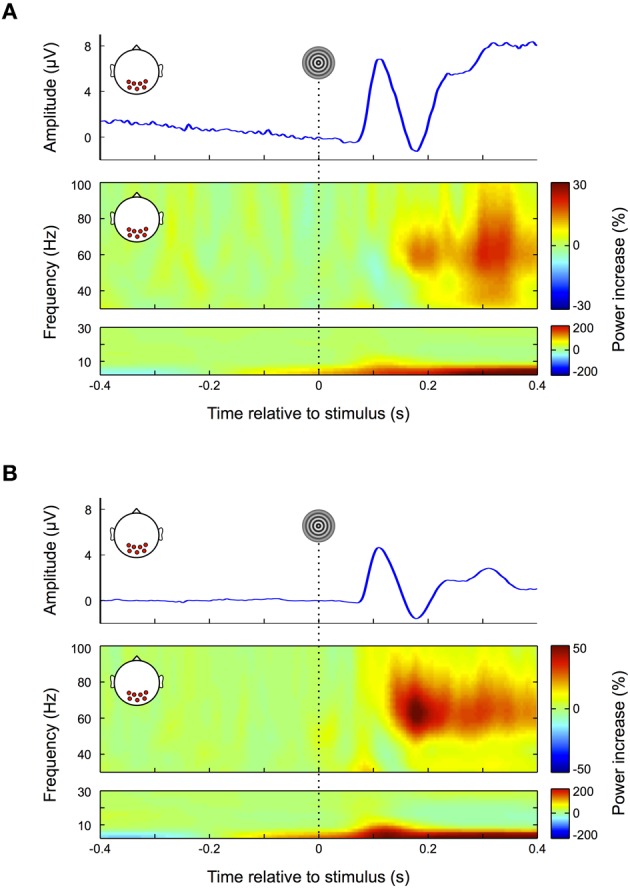
**Visual response. (A)** Nose-referenced data. During fixation trials the average ERP over occipital electrode sites displays a clear visual response (upper panel), i.e., an early transient response, followed by a prolonged increase in amplitude. In the gamma frequency range (30–100 Hz, middle panel) power increases from about 180 ms until the end of the trial. Unlike similar responses in earlier studies at source level the power increase here is not restricted to a defined frequency band over its whole time course but also spreads over the whole gamma range in the later portion of the trial. The power increase in the lower frequency range (0–30 Hz, lower panel) broadens in bandwidth coinciding with the transient response in the ERP. **(B)** Average-referenced data. Similar to **(A)** the ERP displays an early transient and later prolonged response; however, the signal is smoother with lower amplitudes (upper panel). Compared to the nose referenced data, the prolonged response in the gamma frequency band is now more pronounced and confined to a relatively narrow frequency range (middle panel). Similarly the early visual response in the low frequencies is more pronounced.

The results for the nose-referenced data are shown in Figure 3A. The average ERP over occipital electrode sites (top panel) displays a transient visual response followed by a prolonged increase in amplitude, which lasts throughout the remainder of the trial. The slow signal drift observed prior to stimulus onset is most likely due to eye movements in previous trials, which due to the high-pass properties of the recording system cause the signal to slowly drift back toward its initial value. We observed a steady increase in power for frequencies below 5 Hz (Figure 3A, bottom panel), which reflects the general positive trend of the signal over the whole trial (see also Figure 3A top panel). Between approximately 80–160 ms a peak in the alpha range (8–14 Hz) appears to be partially occluded by the lower frequency power increase. Due to its time and frequency range this alpha peak can be attributed to the transient visual response in the ERP. In the gamma band (Figure 3A, middle panel) power increases up to 20% with respect to baseline (<0 ms). In accordance with previous findings the early (150–250 ms) and the late part (380–400 ms) of this response are confined to a frequency range between ~50–80 Hz. However, in the time interval between 250 and 380 ms the bandwidth of the power increase broadens and expands over entire gamma frequency range (30–100 Hz).

As this latter finding differs from earlier studies using MEG, ICA, and invasive animal recordings (Hoogenboom et al., 2006; Fries et al., 2008; Koch et al., 2009) we average-referenced the data to make it more comparable to these reference free methods (Nunez and Srinivasan, 2006). The results are shown in Figure 3B: the slow signal drift in the ERP disappears and the peak amplitude of the early visual response is decreased (Figure 3B top panel). Moreover, the early visual response becomes more pronounced in the low frequencies (<30 Hz) (Figure 3B bottom panel) and extends to the lower gamma frequency range (<40 Hz, Figure 3B middle panel). Similarly the prolonged gamma band response is substantially increased and confined to a well-defined frequency range that is in concordance with earlier studies using source level analysis (Hoogenboom et al., 2006; Fries et al., 2008; Koch et al., 2009). As a result the visually evoked gamma increase now becomes distinguishable from the transient and broadband increase in gamma activity (~30–100 Hz; see also section “Microsaccades”) that is observed in association with fixational eye movements (Fries et al., 2008; Yuval-Greenberg et al., 2008).

### Corneo-retinal dipole movement

**Figure 4:**
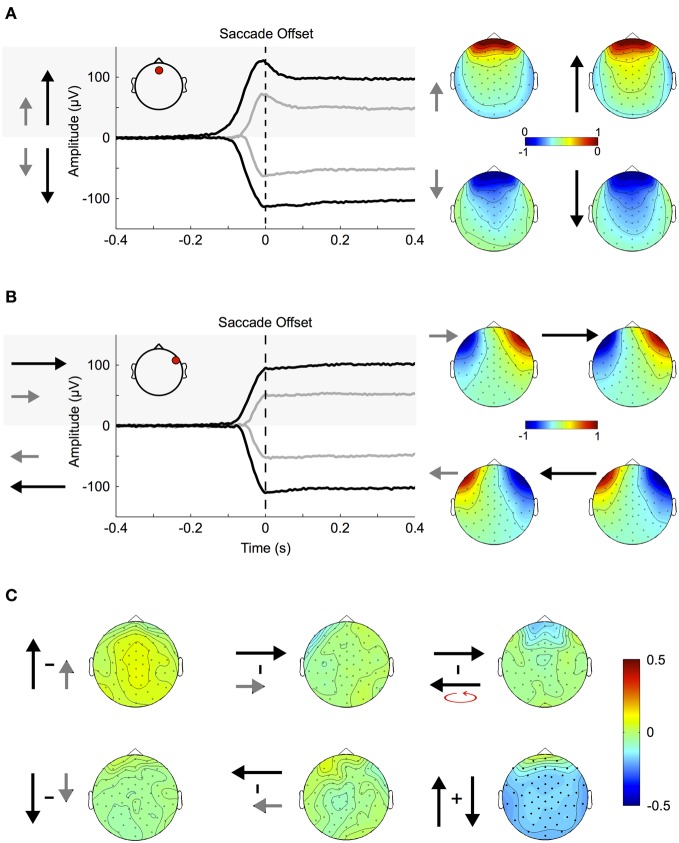
**Corneo-retinal dipole offsets. (A)** Left: ERPs during small (gray traces) and large (black traces) up- and downward saccades as measured at a fronto-central electrode (inset, red circle). The traces show that amplitude and duration of corneo-retinal dipole offsets scale about linearly with saccade size. Right: The normalized topographies during small and large saccades. **(B)** Small and large horizontal saccades. Same conventions and results as in **(A)**. **(C)** Topographic differences between different types of saccades: The normalized topographies of small and large saccades in the same vertical (left column) and horizontal (middle column) direction do not display any significant differences, indicating that the linear relationship between saccade size and signal offset holds for all electrode sites. The same is observed for horizontal movements of the same size but opposite directions (right column, top). In the vertical dimension, however, downward saccades produce a significantly larger offsets than upward saccades of the same size (right column, bottom; significant electrodes marked by bold black dots).

Large ocular movements result in prominent transients and offsets in the EEG signal. These are caused by an orientation change of the eyeball and thus of the corneo-retinal dipole produced between the negatively charged retina and the positively charged cornea. The impact of corneo-retinal dipole changes on the EEG signal is illustrated in Figure 4.

Figure 4A shows the ERPs for small (~11.5°, gray traces) and large (~23°, black traces) vertical saccades measured at a mid-frontal electrode (Figure 4A inset, red circle). Figure 4B shows the same for saccades in the horizontal axis when measured at a right temple electrode (Figure 4B inset, red circle). Depending on whether the cornea moves toward or away from the electrode the signal displays prominent positive and negative offsets, respectively. Within the investigated range, the relationship between movement size and corneo-retinal dipole offset is roughly linear, that is doubling saccade size results in a signal offset of twice the amplitude. Offset topographies are presented in the right column of Figures 4A and B, which illustrate the offset amplitude for different movement directions across the scalp normalized to the mid-frontal (vertical movements) or right temple electrode (horizontal movements). If linearity holds for all electrode sites, the only difference between saccades in the same direction but of different sizes should be in signal amplitude, while the general topographic pattern (i.e., the normalized topography) should stay the same. A direct comparison using a cluster-based permutation test did not reveal any significant differences between the normalized topographies of small and large saccades (*p* > 0.05, Figure 4C, middle and left column). Thus, the relationship between saccade size and signal offset can be considered linear over all electrode sites.

To compare movements of the same size but opposite horizontal directions, we mirrored the topographies of leftward saccades along the midline and subtracted them from the topographies of rightward saccades. Again, we did not observe significant topographic differences (Figure 4C, upper right). In the vertical dimension, however, a change in movement direction results in a significant difference in voltage, which extends over all electrode sites (*p* < 0.05, Figure 4C bottom right).

### Eyelid-induced artifacts

**Figure 5:**
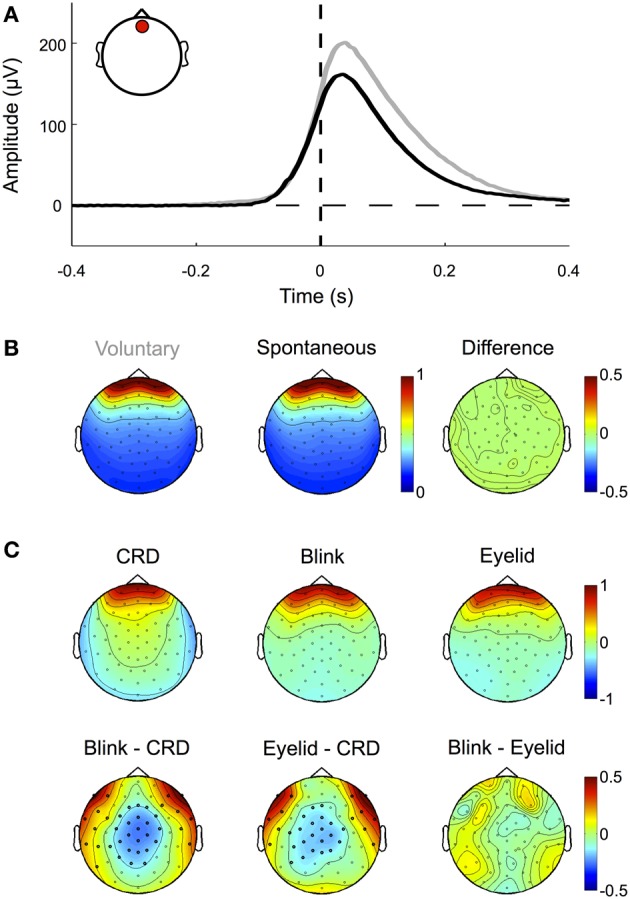
**Eyelid-induced signal changes. (A)** ERP traces for voluntary (gray) and spontaneous (black) blinks measured at a frontal electrode (inset, red circle). Voluntary blinks are of longer duration and result in higher amplitudes than involuntary blinks. Note that blink onset as defined by the eye tracker (time 0, vertical dashed line) corresponds to the point at which the pupil is not visible anymore. The actual eyelid movement already starts around 100 ms earlier, when the signal deflects from the zero line (horizontal dashed line). **(B)** Although spontaneous and voluntary blinks differ in amplitude and duration, they share the same topographic pattern (i.e., the normalized amplitude distribution across the scalp). **(C)** Topographic patterns of corneo-retinal dipole offsets related to upward saccades, blinks, and post-saccadic eyelid movements (upper row) and the differences between them (lower row). Bold black dots indicate electrode sites with statistical significant differences. The results show that corneo-retinal dipole offsets produce a topographic pattern that differs from both, blinks and post-saccadic eyelid movements, while no differences were found between the latter two. This suggests that blinks and post-saccadic eyelid movements are produced by the same electrophysiological source.

Blinks occur spontaneously or can be elicited at will. During blinking the eyelid slides down over the cornea, which is positively charged with respect to the forehead. Thereby the lid acts like a “sliding electrode,” short-circuiting the cornea to the scalp and producing artifacts in the EEG signal (Barry and Jones, 1965; Matsuo et al., 1975; Antervo et al., 1985; Lins et al., 1993a,b). Blink artifacts are easy to identify even in raw data and show a topographic distribution mostly over frontal electrodes. As shown in Figure 5A and demonstrated in earlier studies (e.g., Lins et al., 1993a,b), their amplitude differs (*p* < 0.01) between voluntary blinks (obtained from the pre-experimental procedure) and spontaneous blinks (obtained from excluded trials of the main experiment), while their normalized topographies, and thus their propagation factors onto the scalp, do not (Figures 5A and B).

Although blink artifacts are well-known in cognitive research, other eyelid induced artifacts occurring during and after saccades are often neglected. As illustrated in Figure 4 during upward saccades, the corneo-retinal dipole offset reaches its maximum when the saccade ends (time 0 ms, = fixation onset). However, after this offset change, a smaller second change of opposite polarity can be observed. This latter change exceeds saccade duration and it is known to be produced by eyelid movements that go along with the saccade (Barry and Jones, 1965; Chioran and Yee, 1991). More specifically, saccades are accompanied by ballistic eyelid movements, so called eyelid-saccades, which occur in synchrony with the rotation of the eyeball and therefore are not distinguishable from the corneo-retinal dipole offset in the raw data. In other words, during upward saccades for instance, eyelid and eyeball move upwards with approximately the same speed. However, after the termination of both, eye- and eyelid saccades, the eyelid continues to slide more slowly for another 30–300 ms and produces a signal change that is observable particularly after upward saccades (Barry and Jones, 1965; Becker and Fuchs, 1988). In contrast to eyelid artifacts that co-occur with the saccade, this post-saccadic signal change, although well-established in opthalmology, is rarely mentioned or described in relevant publications in cognitive sciences (cf. Keren et al., 2010; Dimigen et al., 2011).

To confirm previous findings and to investigate how this post-saccadic eyelid artifact affects our data, we compared the difference between the signal amplitude measured at a frontal electrode (see inset Figure 5A) at the time of saccade offset and the signal amplitude at the same electrode but 100 ms later, when the eyelid-induced signal change has reached its final level (as for example seen in Figure 4A). A Two-Way repeated-measures ANOVA (three movement types: periphery to center, center to periphery and periphery to periphery; four movement directions: up, down, left, and right) revealed that amplitude differences between saccade offset and eyelid offset depend on both, movement type (*F* = 25.63, *p* < 0.001) and movement direction (*F* = 42.47, *p* < 0.001). We observed significant amplitude differences between long and short saccades (*p* < 0.001) and found that eyelid-induced amplitude changes were significantly larger (*p* < 0.01) for upward saccades than for all other movement directions. Together with the finding that there is an interaction between type and direction of the movement (*F* = 2.55, *p* < 0.01) this confirms earlier reports that eyelid-induced amplitude changes are most prominent after upward directed eye movements (Barry and Jones, 1965; Becker and Fuchs, 1988; see also Figure 4A).

Next, we compared the normalized topographies of blink artifacts, corneo-retinal dipole offsets and post-saccadic eyelid artifacts. As blink- and saccade-related eyelid artifacts are produced by the same mechanism, namely the eyelid sliding over the cornea, their activity projects to the scalp with the same topographic pattern (Figure 5C). Topographies related to corneo-retinal dipole offsets on the other hand differ from both, those of blinks and those of post-saccadic eyelid movements, with significant differences clustered around the central and fronto-lateral regions (*p* < 0.05, Figure 5C). Effectively these results indicate that eyelid induced artifacts and corneo-retinal dipole offsets arise from two different electrophysiological mechanisms that cannot be modeled as a single source.

### Spike potential

**Figure 6:**
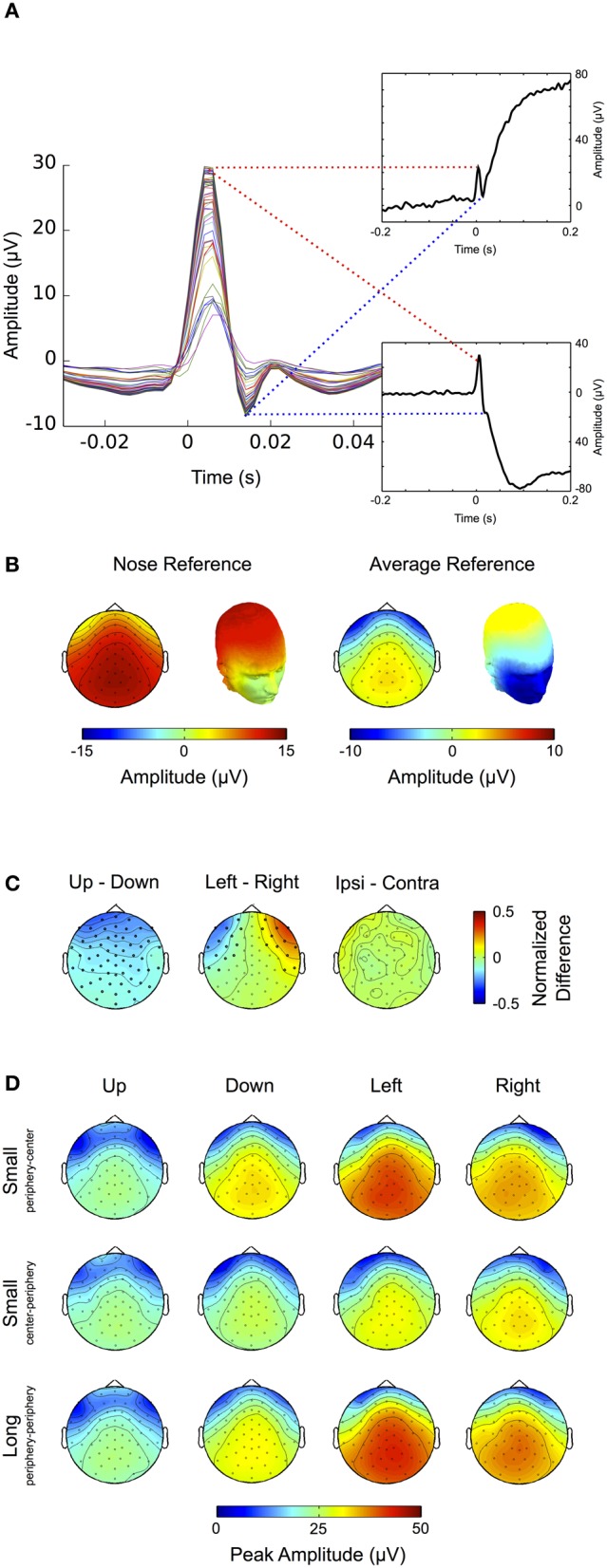
**Saccadic spike potential. (A)** After removal of the corneo-retinal dipole by high pass filtering the data at 30 Hz, the spike potential displays a biphasic shape at all scalp electrodes (colored traces). Without filtering the ERP traces for up- (upper inset) and downward (lower inset) saccades show that the negative deflection of the spike potential is largely occluded by the manifold larger corneo-retinal dipole offset. **(B)** Average referencing reduces the amplitude of the spike potential at scalp electrodes and leads to increased negativity around the eyes. **(C)** Differences of topographic patterns for spike potentials related to saccades in the same axis but opposite directions. Comparing spike potentials related to up- and downward saccades of the same size results in significant amplitude differences at almost all scalp electrodes, indicating that downward saccades produce higher spike potential amplitudes. The difference between left and rightward saccades yields significant values at electrode sites close to the eyes. However, no significant differences were found between spike potentials related to ipsi- and contralateral saccades, suggesting that the topographic differences for saccades with the same size and along the same axis may be not so much related to the spike potential's amplitude itself but rather to eye position (i.e., the direction of the corneo-retinal dipole) before saccade onset. **(D)** Spike potential topographies for different saccade directions (columns) and sizes (rows). A two-way ANOVA reveals that peak amplitudes of the spike potential are higher for down- than for upward saccades while they are not significantly different between left and rightward saccades. Smaller saccades from the periphery to the center result in higher spike potential amplitudes than saccades of the same size but performed from the center to the periphery. Surprisingly saccades from the periphery to the center do not show significant differences to long saccades (i.e., saccades performed from the periphery across the center to another peripheral location). This indicates that the peak amplitude of spike potentials may depend on initial eye position, rather than on saccade size.

In early studies the saccadic spike potential has been described as a “monophasic potential appearing just before saccade onset” (Thickbroom and Mastaglia, 1985). However, concordant with earlier studies (Riemslag et al., 1988; Nativ et al., 1990; Keren et al., 2010), high-pass filtering the data at 10 Hz and thereby removing corneo-retinal dipole offsets reveals that the saccadic spike potential actually displays a biphasic waveform, starting around 5 ms prior to saccade onset and consisting of a larger positive deflection followed by a smaller negative deflection (Figure 6A). Note that in the present study, as compared to the above-mentioned ones, the polarity of the waveform is inverted. This is because we describe the properties of the saccadic spike potential at scalp electrodes rather than at EOG channels (Keren et al., 2010) and because we used a nose reference, while other studies referenced to an electrode attached to the temporal bone (Thickbroom and Mastaglia, 1985; Riemslag et al., 1988). Since in the unfiltered data the negative deflection of the saccadic spike potential is largely occluded by the corneo-retinal dipole offset (Figure 6A, insets) the following analysis will only focus on the positive peak of the saccadic spike potential.

To investigate amplitude and topography of the saccadic spike potential for saccades of different sizes and directions, we relate the data to a 5 ms baseline before the onset of the spike potential (i.e., −10 to −5 ms relative to saccade onset). Figure 6B shows the topography of the positive peak of the spike potential averaged over all saccade directions. When the data is average-referenced, the topographic pattern stays the same while the magnitude is shifted from central-parietal electrodes to electrode sites surrounding the eyes (Figure 6B, cf. Keren et al., 2010). For the remainder of the analysis of saccadic spike potentials we used nose-referenced data exclusively.

To compare the topographic pattern of spike potentials between eye movement directions we normalized the respective topographies to the maximum amplitude over all saccade directions. The difference between spike potentials accompanying up- and downward saccades shows that during upward saccades at almost all electrodes the measured potential is significantly lower (*p* < 0.05) than during downward saccades (Figure 6C, left). A possible explanation is that at the onset of a downward saccade the eye, and thus the positive pole of the corneo-retinal dipole, is directed upward, consequently leading to a more positive topography as compared to the onset of upward saccades where the positive pole of the corneo-retinal dipole is initially directed downwards. This is also supported by the observation that topographic differences between left- and rightward saccades become significant (*p* < 0.05) at electrodes around the eyes and thus close to the corneo-retinal dipole (Figure 6C, middle). We found no differences between the topographic patterns of ipsi- and contralateral saccades (i.e., the topography of leftward saccades versus the horizontally flipped topography of rightward saccades, Figure 6C, right).

Next we compared how the amplitude of saccadic spike potentials changes depending on the type of eye movement. Figure 6D shows the respective amplitudes and topographies for different saccade sizes, directions and initial eye positions, that is up-, down-, left- and rightward saccades performed from the periphery to the center, from the center to the periphery and from the periphery to the opposite peripheric region (i.e., long saccades). For each of these classes we determined the highest peak at central-parietal electrodes during a time period ranging from −5 to +10 ms relative to saccade onset. A Two-Way repeated-measures ANOVA revealed that the amplitude of the saccadic spike potential depends on both direction (*F* = 13.05, *p* < 0.001) and type of the movement (*F* = 29.06, *p* < 0.001). It significantly differs between vertical and horizontal saccades (*p* < 0.05, Bonferroni corrected) and movements within the vertical dimension (i.e., upward and downward saccades, *p* < 0.001), but not between left- and rightward saccades. The amplitude of saccadic spike potentials has been shown to increase non-linearly with saccade size up to 1.28° before reaching its final maximum (Armington, 1978). However, as saccade sizes in our experiment exceeded this threshold, we did not expect any amplitude differences between spike potentials related to long (~23°) and short (~11.5°) saccades. But interestingly the amplitudes observed for short saccades from the center to the periphery were significantly different from those accompanying long saccades (*p* < 0.001). This difference, however, cannot be attributed to saccade size, as it was not observed between long saccades and short saccades from the periphery to the center.

To check whether this rather surprising finding could be a result of filter-induced slow signal drifts in the recording, as described above (i.e., saccades to peripheral screen positions are generally larger and therefore might produce larger drifts when the signal returns back to baseline), we repeated the analysis after high pass filtering the data at 10 Hz, and in this way eliminating such drifts. However, using filtered data yielded the same results as using unfiltered data. This suggests that for saccades larger than ~11.5° the amplitude of the saccadic spike potential depends on the initial eye position, rather than on saccade size per se.

### Microsaccades

Microsaccades are small eye movements that occur during fixation. They distinguish themselves from regular saccades not only in amplitude but also in that they are performed involuntarily.

**Figure 7:**
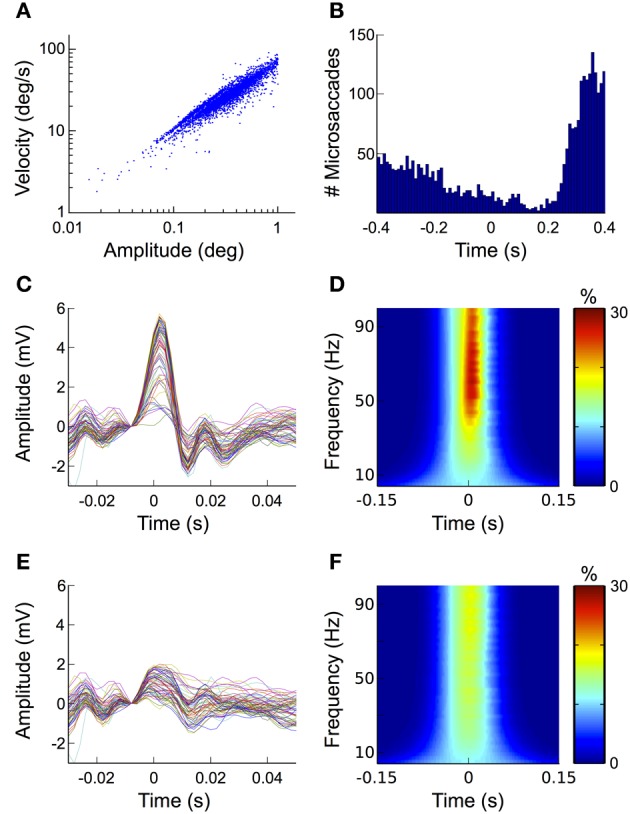
**Microsaccades. (A)** Mainsequence. Plotting microsaccade amplitudes against microsaccade velocities results in a straight line on a log/log scale (i.e., the mainsequence). This relationship is a signature for ballistic eye movements, thus confirming the physiological origin of the microsaccades detected here. **(B)** Histogram of the microsaccade distribution over all fixation trials. After stimulus onset the frequency of microsaccades decreases, followed by a rebound starting at around 200 ms and peaking 370 ms after stimulus onset. **(C)** ERP aligned to microsaccade onset. At scalp electrodes microsaccades display a similar biphasic pattern as the saccadic spike potential, suggesting that the most prominent contribution of microsaccades to the signal measured on the scalp is produced by spike potentials going along with eye movement. **(D)** Time-frequency signature of microsaccades. The sharp peak of the microsaccade-related spike potential results in a transient broadband power burst that spans over the entire gamma frequency range (30–100 Hz). **(E)** Reduction of microsaccade-related artifacts in the time domain. The ICA-based correction procedure proposed here diminishes the microsaccade-related spike potential to about one third of its original amplitude. **(F)** Reduction of microsaccade-related artifacts in the frequency domain. Corresponding to what was observed for the ERP, the correction procedure substantially reduces the spike potential-related frequency signature in the gamma band.

In the fixation condition we detected a total of 4252 microsaccades over all trials and subjects, which corresponds to an average of 1.07 microsaccades per trial. Note that although sometimes other or additional criteria (e.g., larger or smaller amplitude range, involuntary performance etc.) are applied, here we defined all eye movements with amplitudes between 0.1 and 1° of visual angle as microsaccades. To ensure that the detected movements were indeed microsaccades and not noise in the eye movement recordings, we investigated the relationship between their amplitude and velocity. In agreement with previous descriptions (Martinez-Conde et al., 2004), the microsaccades detected in our data follow a linear relationship when plotted on a log-log scale (i.e., the “main sequence,” Figure 7A).

Microsaccades typically occur with a frequency of 1–2 Hz (Engbert, 2006). The probability of their occurrence, however, is not equally distributed over time but follows a typical temporal pattern with respect to stimulus onset: after stimulus presentation their rate initially decreases to its minimum at around 150 ms. Thereafter it rapidly increases again reaching its maximum at about 350 ms (Engbert and Kliegl, 2003; Dimigen et al., 2009). When we investigated the distribution of microsaccades over all fixation trials and all subjects, we found that it matched this pattern very closely (Figure 7B).

To study the impact of microsaccades on the EEG signal, we segmented the data into epochs time-locked to microsaccade onset. The ERP of these epochs displays the same biphasic waveform as the saccadic spike potential of larger saccades, although its amplitude is substantially smaller (Figure 7C). In the time-frequency analysis of our data this pattern manifests itself as a transient burst between 40 and 100 Hz (Figure 7D), which is in concordance with earlier studies (Yuval-Greenberg et al., 2008; Keren et al., 2010). Altogether these findings confirm, that microsaccade-induced confounds in the EEG are mainly caused by spike potentials occurring at microsaccade onset, while orientation changes of the corneo-retinal dipole only play a minor role.

### Artifact correction

In order to evaluate the efficiency of different approaches to eye artifact correction we compared the performance of a two and a three component regression model (Elbert et al., 1985; Schlögl et al., 2007) and ICA (Jung et al., 2000).

In order to objectively distinguish eye movement-related from non-eye-movement-related ICs we employed a selection procedure based on eye tracking information. More specifically, we compared every IC's activation during saccade and fixation periods. If the respective IC displayed a higher activation during saccades than during fixation, it was classified as eye movement-related and rejected from the data (see section “Independent Component Analysis” and Figure 2 for more details). Following this procedure we found between 3 and 11 artifactual ICs for each subject, accumulating to a total number of 74 (out of 896) rejected ICs.

To test how well the automated selection algorithm performed in comparison to visual selection by experienced researchers, we asked two independent experts to tag all ICs in our data that they considered as eye movement-related. The classification was done in three steps: (1) we asked both experts to identify eye movement-related ICs solely based on their respective topographies. Over all subjects and components, Expert 1 classified 36 ICs as eye movement-related, while Expert 2 identified 41 ICs as related to ocular artifacts. (2) Next we asked the experts to repeat their evaluation but this time based on both, the ICs' topographies and their activations during three example trials, each of which contained an eye movement in a different direction (up, down, and left). Given the additional information about the ICs' activations, Expert 1 rated 16 additional ICs as eye movement-related. Similarly, Expert 2 tagged 16 more ICs as eye artifacts but additionally revised his previous judgment about four components, which he removed from his selection. Now, in total 52 ICs were tagged by Expert 1 and 53 ICs by Expert 2. Notably, apart from the one additional IC identified by Expert 2, both experts' selection of eye movement ICs was identical. This strongly suggests that including IC activations as a decision criterion leads to a substantial improvement in effectiveness and reliability of IC classification. (3) In a last step we determined all ICs that had a variance ratio (i.e., variance of saccade intervals vs. variance of fixation intervals; see section “Independent Component Analysis” and Figure 2) above one and that the respective expert had not tagged as eye movement-related before. For these ICs, next to their topographies, we now provided the activations during all available saccade trials and again asked the experts to classify them as either eye movement-related or unrelated. Now having a large number of trials available, Expert 1 tagged another 16 ICs and Expert 2 another 15 ICs. Thus, in total both experts rated 67 ICs out of 896 as eye movement-related and their assessment was now perfectly congruent.

Out of the 67 ICs identified by human scorers, 64 ICs were also detected by the automated selection procedure. Additionally the algorithm tagged 7 ICs that did not conform to the experts' assessment. However, as 6 of these 7 ICs were from only one subject and their topographies peaked in neighboring regions of the scalp, we assume that these 6 ICs may have entered the selection due to unusual activity and/or noise in this particular subject's data.

**Figure 8:**
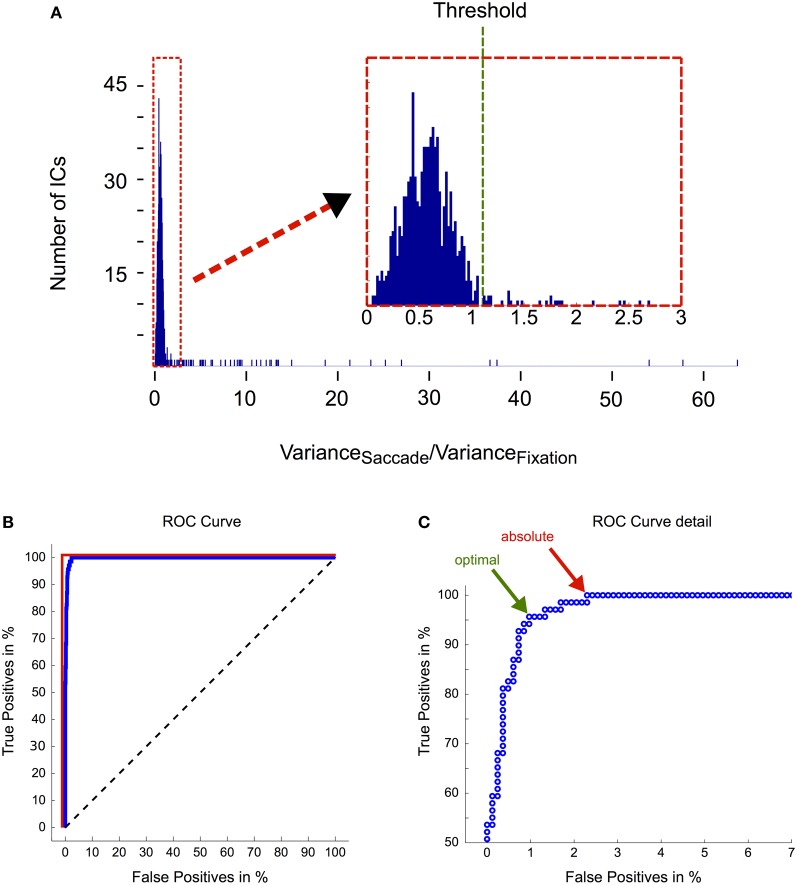
**Evaluation of the IC selection procedure. (A)** Distribution of saccade/fixation variance ratios. The magnification in the inset illustrates that the ratios are not clearly bimodally distributed. Therefore the threshold that optimally separates eye movement-related ICs from non-ocular ICs is difficult to determine. Based on heuristics, that is after inspecting IC activations that had a ratio above one and that we considered likely to be eye movement-related, we set the threshold to a ratio of 1.1. **(B)** ROC analysis. ROC curves are graphical illustrations of how well two different classes (here: eye movement-related ICs vs. non-eye movement-related ICs) can be separated depending on the threshold of the discrimination criterion (here: the ratio between the variance in saccade and fixation intervals). Each point on the blue curve represents the saccade/fixation variance ratio of one IC starting with the highest (63.71) in the lower left corner and ending with the lowest (0.04) in the upper right corner. If ICs could be unequivocally separated into being eye- or non-eye-related solely based on their variance ratio, lowering the threshold would include more and more eye movement ICs (as determined by expert tagging) until a true positive rate of 100% is reached. Subsequently by further lowering the threshold more and more non-eye movement-related components would be included in the selection. In this case the blue curve would be identical with the red line. Conversely, if the variance ratio would not provide any information about the IC's relation to eye movements, the blue curve would follow the black dashed line. The area under curve (AUC), which is obtained by computing the area between the blue and the black dashed line, quantifies how well eye movement-related ICs are separated from other ICs only based on their saccade/fixation variance ratio. An AUC value of 1 indicates perfect discrimination and a value of 0.5 indicates random performance. Here we observed an AUC value of more than 0.99. **(C)** ROC curve detail. The green arrow indicates the the optimal threshold for separating the ICs into two classes. It is given by the point at which further lowering the threshold would include more false positives than true positives. Here it has a value of 1.11 and is thus very close to our pre-determined threshold. The red arrow indicates the threshold at which all eye movement-related ICs are included in the selection. The corresponding ratio is 0.99.

For further quantification of the algorithm's efficiency and to test the adequacy of the pre-determined ratio threshold of 1.1 (Figure 8A), we performed a receiver operating characteristic (ROC) analysis (Figure 8B; see figure captions for a description of the method), where ICs tagged by human experts served as reference (true positives). We observed an area under curve (AUC) value of more than 0.99, which indicates that eye movement-related ICs are almost perfectly separable from other ICs solely based on their saccade/fixation variance ratios. The optimal threshold for this separation is defined by the point at which further lowering the threshold would include more false positives than true positives and is indicated by the green arrow in Figure 8C. The respective saccade/fixation variance ratio at this point is 1.11 and the following one (i.e., the first suboptimal threshold) has a value of 1.06. This means that the pre-determined threshold of 1.1 turned out to be chosen optimally for the dataset presented here.

Since it is not possible to measure uncontaminated neural activity with EEG, the validation of correction procedures is always suboptimal (Croft and Barry, 2000a,b). Simple inspection of corrected raw data by expert scorers as used elsewhere (Jung et al., 2000; Joyce et al., 2004; Schlögl et al., 2007) might be insufficient, as small residuals of artifacts may be not noticeable in the raw signal. Averaging over many trials however increases the signal-to-noise ratio and thus facilitates the detection of such residuals. For this reason we evaluated correction performance based on the ERP and the time-frequency response, both time-locked to the saccade.

**Figure 9:**
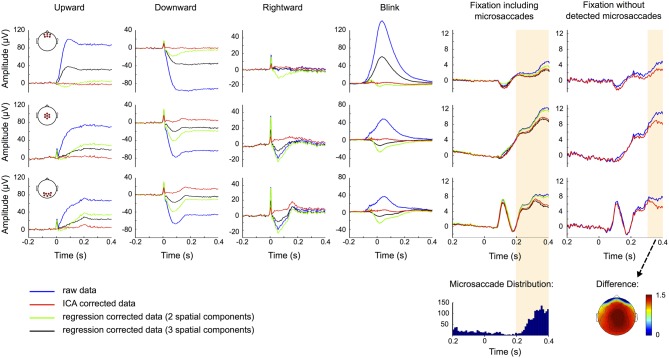
**ERP correction.** ERP traces for uncorrected data (blue), data corrected based on two (green) and three (black) component regression models and ICA corrected data (red). Rows show the ERPs measured at frontal (top), central (middle), and occipital (bottom) scalp locations and the red circles in the head plots on the left indicate the respective electrode sites. Columns correspond to up-, down-, and rightward saccades, blinks and fixation trials with and without detected microsaccades, respectively. Time 0 represents saccade and blink onset as determined by the eye tracker and stimulus presentation in fixation trials. The traces suggest that regression tends to over- or under-correct the data. ICA on the other hand efficiently removes corneo-retinal dipole offsets and eyelid artifacts, while the visual response is still clearly seen in occipital channels. However, ICA fails to entirely remove the spike potential at saccade onset but still reduces it substantially. In the fixation condition including microsaccades the raw and the ICA corrected data display significant differences (shaded area) in the time interval after 198 ms, but the distribution of microsaccades over all trials (bottom row) suggests that a significant portion of these differences can be attributed to the reduction of microsaccade-related artifacts. This is supported by the observation that in fixation trials without detected microsaccades, the difference between raw and ICA corrected data is smaller and only becomes significant after 278 ms. The topography of this difference resembles the one of the saccadic spike potential and may therefore be related to undetected microsaccades.

The results of ERP correction by using 2 (green traces) and 3 (black traces) model regression and the proposed ICA-based procedure (red traces) are shown in Figure 9. Artifact correction by regression fails to completely remove the offset produced by eye movements or blinks. In any case, apart from spike potential suppression, the correction by the regression model with two spatial components performs better than the one with three spatial components. This probably reflects an excessive introduction of correlated neural activity to the EOG measurements due to the use of temporal channels for the calculation of the axial component of the model. Because regression coefficients were obtained in a period including both eye movements and blinks, regression fails to entirely remove offsets because the regressors that are fit to the data are based on both, corneo-retinal dipole offsets and eyelid artifacts. As a result the weights that are assigned to each electrode for all movement directions constitute a compromise between these two artifacts. For more posterior channels (Figure 9, 2nd and 3rd row) this results in over-correction for blinking and under-correction for saccades, as blink peaks propagate less to the back than saccade offsets do (see Figure 5C).

ICA on the other hand independently removes both, corneo-retinal dipole offsets and eyelid artifacts, and thus accounts for the fact that their relative contribution to the measured signal varies for different movement directions. However, except for upward saccades ICA failed to entirely remove the spike potential at saccade onset, but nevertheless reduced it by about 75% depending on saccade size and direction (Figure 9, red traces). The visual response is still clearly seen in occipital channels but due to variations in saccade duration it does not display its distinctive shape (see Figure 3) but manifests itself as a flat and broad peak at around 180 ms.

To check whether the procedure might over-correct the data and thus remove neural signals along with eye movement artifacts, we compared the raw and the corrected data during fixation trials (Figure 9, 5th column). We observed a significant difference (*p* < 0.001, shaded area) between ERPs of raw (blue traces) and ICA corrected data (red traces), comprising a time interval from 198 ms after stimulus onset to the end of the trial. However, the distribution of microsaccades over all trials (Figure 9, 5th column bottom) and the observation that after correction microsaccade-related spike potentials are reduced to about one third of their initial value (Figure 7E), suggest that a significant part of this difference can be attributed to the reduction of microsaccade-related artifacts, rather than to removal of neural activity. To test this assumption we repeated the analysis with only those trials in which we did not detect any microsaccades. As seen in the rightmost column in Figure 9 the difference between raw (blue) and ICA corrected data (red) is now smaller, especially in central and occipital regions, and only becomes significant 278 ms after stimulus onset, which is 80 ms later than what we observed in trials including microsaccades. The topography of the remaining difference strongly resembles the topography of the saccadic spike potential. We therefore conclude that a large part of this difference may be due to mircosaccades that are still present in the data but were not detected in our eye tracking data. Additionally, analogous to average referencing, ICA correction may reduce global noise and power (see also Figure 2). This may also be the reason why during fixation trials the regression model with a third spatial component spanning across a large part of the brain is more similar to the ICA corrected than to the raw data (Figure 9, 5th column).

In summary, rejecting ICA components based on eye tracking information resulted in complete removal of corneo-retinal dipole offsets and eyelid artifacts from the ERP. It also reduced confounds related to saccadic spike potentials to a very large extent, without substantially affecting signals from neural sources.

**Figure 10:**
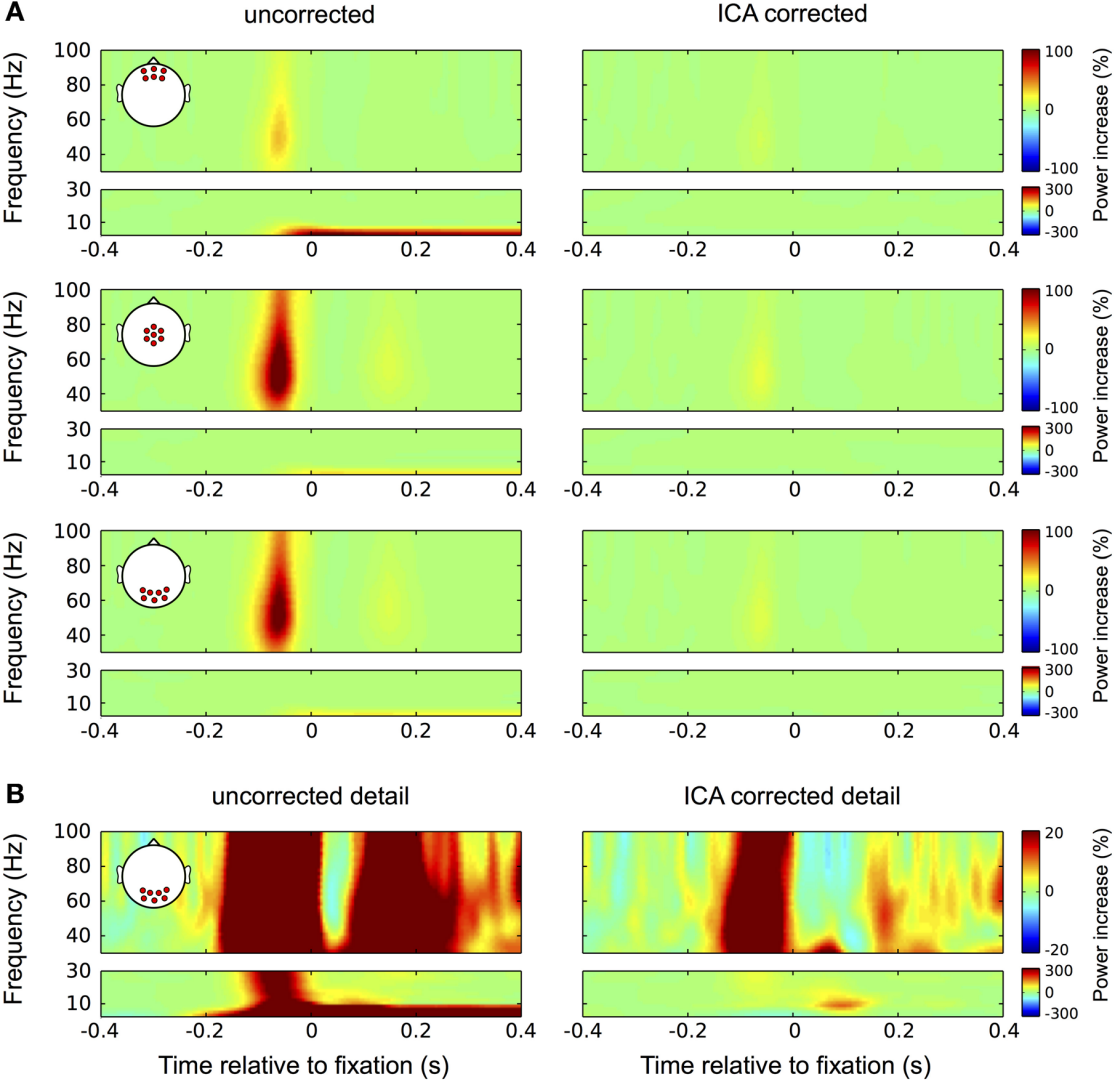
**Correction in the time-frequency domain. (A)** Correction of eye movement artifacts in the high (larger boxes) and low (smaller boxes) frequency ranges at frontal (top panel), central (second panel), and occipital (bottom panel) electrode sites. Left column: In the high frequencies (30–100 Hz) the uncorrected data display the typical broadband gamma burst-related to the spike potential at saccade onset. Corresponding with the topography of the spike potential this burst is most pronounced at central and occipital channel locations. As a result of small correctional saccades, a similar but much weaker burst is observed about 180 ms after fixation onset. In the low frequencies (<30 Hz) the corneo-retinal dipole offset leads to a prolonged increase in power below 10 Hz, which is starting at saccade onset and manifests itself most prominently at frontal electrode sites. Right column: In concordance with the observation for ERP data, ICA correction significantly reduces the spike potential-related gamma burst and completely removes the power increase related to corneo-retinal dipole offsets. **(B)** Correction of eye movement artifacts at occipital electrode sites on a more detailed color scale. Left column: The second broadband burst in the gamma range, which is caused by correctional saccades occludes the prolonged visual response we found earlier during fixation trials. Similarly, with the more detailed color scale confounds produced by the spike potential are also observed in the low frequency range and together with the corneo-retinal dipole-related power increase, the early visual response that is observed in the ERP is largely occluded. Right column: The more detailed color scale confirms that ICA completely removes corneo-retinal dipole induced power changes, while the spike potential still visible for both, task-related and correctional saccades. However, the removal of corneo-retinal dipole offsets and reduction of the spike potential renders the visual responses clearly visible in both the low and high frequencies.

Nevertheless, confounds that are not strictly time-locked to the saccade or that are restricted to a certain frequency range may go unnoticed in the ERP. Therefore we evaluated eye tracking based IC rejection also with respect to its efficiency in the frequency domain. The results are seen in Figure 10: in the high frequencies (30–100 Hz) the uncorrected data (left column) display the typical broadband gamma burst-related to the spike potential at saccade onset. Consistent with the topography of this spike potential this burst is most pronounced at central and occipital channel locations. Additionally, as a result of small correctional saccades, a similar but much weaker burst is observed about 180 ms after fixation onset. In frequencies below 10 Hz the corneo-retinal dipole offset leads to a general power increase, which is most pronounced at frontal electrode sites. In concordance with the observation for ERP data, ICA correction (left column) completely removes power changes related to corneo-retinal dipole offsets and eyelid artifacts, and reduces the high-frequency correspondent of the saccadic spike potential by about 85% (Figure 10A). Note that in concordance with earlier studies (Keren et al., 2010) a similar reduction was also observed for microsaccadic spike potentials (Figure 7F).

Investigating the data at occipital electrode sites on a lower color scale provides a more detailed view on the properties of eye movement confounds and the impact of the correction procedure (Figure 10B). In the uncorrected data (left column) the broadband gamma burst of the saccadic spike potential extends into the low frequency range and together with the power increase related to corneo-retinal dipole offsets, covers the early transient visual response that was observed in the ERP. Additionally, the second broadband burst largely occludes the later visual response in the gamma range (see Figure 3). In the corrected data on the other hand, both, the early transient (after 70 ms) and the later prolonged visual response (after around 200 ms) become clearly visible in the low and high frequencies, respectively (Figure 10B, right column).

**Figure 11:**
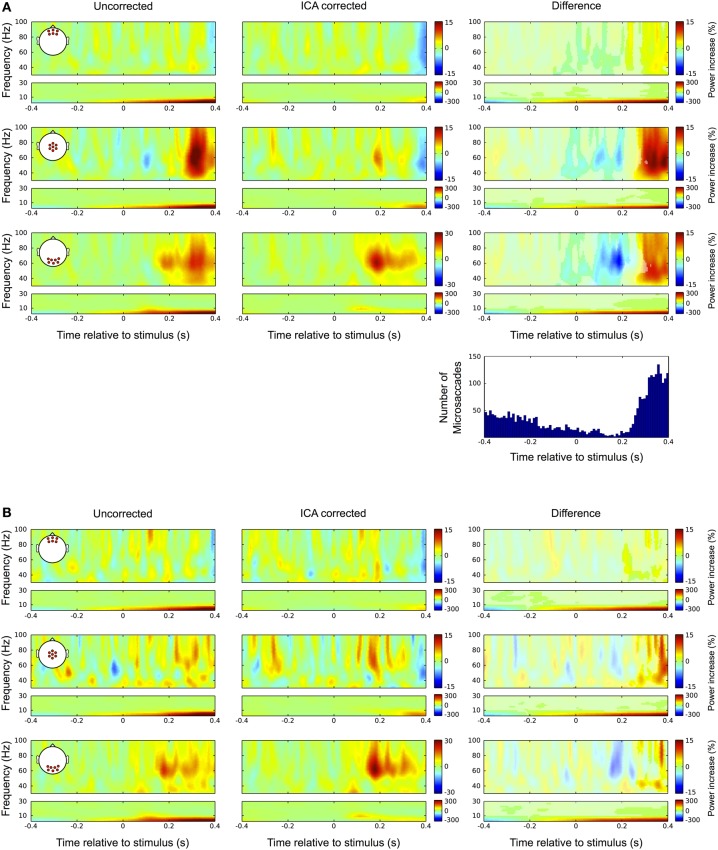
**Differences between uncorrected and ICA corrected time frequency data during fixation trials. (A)** Same conventions as in Figure 9. Left column: In the uncorrected data a prominent broadband gamma power increase is observed at central electrode sites. Occipital channels display prolonged activity in the gamma range corresponding to the late visual response as described in Figure 3. Middle column: In the corrected data the prolonged visual gamma response is confined to a frequency band between approximately 50 and 80 Hz and its peak now also visible in central channels. The low frequencies reveal a distinct peak at around 9 Hz corresponding to the early transient response in the ERP. Right column: Comparing the difference between corrected and uncorrected data with the distribution of microsaccades indicates that the significant portions of the observed differences (i.e., bins in original color; non-significant bins are gray shaded) follow the pattern of microsaccade distribution very closely. The negative gamma power difference after stimulus onset results from a drop in microsaccade rate relative to the pre-stimulus interval, which here serves as the baseline. Then coinciding with the increase in microsaccade rate after 250 ms the difference in gamma power substantially increases. **(B)** In trials without detected microsaccades the largest part of the broadband gamma power increase disappears and smaller differences between raw and ICA corrected data only become significant after 300 ms. Again these residual differences display the spatial and spectral signatures of the saccadic spike potential and therefore are likely the result of undetected microsaccades.

Again, we checked whether the correction procedure affects the signal during fixation trials, as in the absence of eye movements there should not be any discrepancies between the raw and the ICA corrected data. Figure 11A shows the results. In the uncorrected data (left) we observed a prominent gamma power increase at central electrode sites starting at around 250 ms after stimulus onset. That corresponds to the time and frequency range of the bandwidth increase of the visual gamma band response in occipital channels, which was described above (Figure 3). In the corrected data this broadband gamma increase disappears and the visual response is confined to a frequency band between 50 and 85 Hz similar to what was observed in the average-referenced data and reported in earlier studies. Moreover, the peak of this response is now also visible in central channels. In the lower frequency range the corrected data reveal a distinct peak at 9 Hz, corresponding to the early transient response in the ERP. Comparing the difference between corrected and uncorrected data (right column) with the distribution of microsaccades indicates that the significant portions of the observed differences (original color, non-significant bins are grey shaded) follow the pattern of the distribution very closely. The power drop-off in gamma band after stimulus onset seems to be caused by a drop in microsaccade rate relative to the pre-stimulus interval, which here serves as the baseline. The rebound of microsaccade rate after 230 ms coincides with a substantial increase in gamma power, again indicating that the observed differences are caused by miniature eye movements during fixation. Like with the ERP data before, we tested this presumption by repeating the analysis with only those trials in which we did not detect any microsaccades. The results are shown in Figure 11B: as compared to trials with microsaccades, the broadband power increase after 250 ms at central and occipital channels largely disappears, thus supporting the hypothesis that it was caused by microsaccadic spike potentials. We still find differences between the raw and the ICA corrected data, but now being much smaller and starting later at around 300 ms after stimulus onset. Analogous to our observations in the ERP data we conclude that these differences are mainly caused by undetected microsaccades, rather than by removal of neural activity. This is strongly supported by the frequency signature and scalp distribution of the residual difference, which very closely resemble the ones that we observed for spike potentials and microsaccades, respectively.

## Discussion

We were able to confirm that eye- and eyelid movement-related artifacts arise from several independent mechanisms and showed that their relative contribution to the measured EEG signal depends on the type of eye movement.

We evaluated the performance of ICA and regression-based eye artifact correction methods. As using ICA for artifact rejection generally poses the problem to objectively differentiate between ICs related to artifacts and ICs related to neural activity, we devised a selection procedure that identifies eye artifact related ICs based on eye tracker information. Rejecting these ICs from the data resulted in complete removal or significant reduction of the above-described eye- and eyelid movement artifacts, while leaving the relevant signal emerging from neural sources intact. In contrast, both regression models tested here resulted in suboptimal artifact correction.

Below we will review our findings in the light of previous studies of eye movement artifacts and discuss the resulting implications for artifact correction and data analysis. We discuss why ICA is in principle suited to extract artifacts arising from multiple sources and why regression is likely to fail under these circumstances. Finally we consider neural activity that goes along with eye movements and therefore may have an impact on the data despite artifact correction.

### Eye- and eyelid movement artifacts

#### Eyeball rotation artifacts

Eyeball rotations induce signal changes that depend on both, movement size and direction. The polarity of these changes in turn depend on the orientation of the corneo-retinal dipole, whose axis closely follows the direction of gaze. The corneo-retinal dipole itself is produced by the ionic gradient between the apical and basal surfaces of the retinal pigment epithelium (for a review of the underlying physiology see Arden and Constable, 2006). However, even when the retina is surgically removed, signal changes related to eyeball rotation can still be recorded. Therefore it is probable that other sources also contribute to the generation of the dipole, like for instance potential differences that occur exclusively across the cornea or between the blood and the intraocular fluids (Pasik et al., 1965).

For any given movement direction, the amplitude of the corneo-retinal dipole offset changes roughly linearly with saccade size. However, the magnitude of the corneo-retinal dipole itself is far from static. It has been shown that the potential between cornea and retina changes with illumination (Miles, 1940; Arden and Kelsey, 1962). This change may occur on a time scale ranging from a few milliseconds to several hours. For instance, illumination of a dark-adapted eye produces a rise in potential that peaks after 10–15 min (Lins et al., 1993a). The respective peak amplitude depends on both, the intensity of the light and the duration of the dark adaptation and it is followed by a damped oscillation that continues for hours (Arden and Kelsey, 1962). Next to other reasons (cf. Dimigen et al., 2011), these fluctuations in response to illumination changes constrain the utilization of the EOG as an absolute measure for gaze position, because a reliable estimation would require recalibration about every 10 s (North, 1965). However, changes in the standing potential do not preclude the correction of corneo-retinal dipole-related artifacts since the propagation of the potential through the volume conductor is independent of its intensity (Girton and Kamiya, 1973).

More problematic in this respect is the fact that eyeball rotation can be accompanied by translational or torsional movements of the eye. Such eyeball displacements impede accurately modeling the potential difference between cornea and retina as a single dipole, which in turn leads to difficulties in estimating a single set of scalp propagation factors for a given dipole orientation. Still, in the present study, we found that saccades of different sizes but with the same orientation share the same topographic pattern and thus the same propagation factors of corneo-retinal dipole-related artifacts onto the scalp. This implies that for the movements investigated here, possible displacements of the eyeball do not have significant impact on the measured signal. We also observed that movements of opposite orientation in the horizontal axis do not only result in the same (but mirror reversed) propagation factors but that they also project to the scalp with the same amplitude. Although we found a global difference in amplitude between up- and downward movements, the similarity of their topographic patterns suggests that this is because the reference is not located symmetrical with respect to the plane of eye rotation, rather than because of a difference between their respective propagation factors.

#### Eyelid artifacts

Artifacts emerging from blinks, eyelid saccades, and post-saccadic eyelid movements are produced by the same principal mechanism, namely the eyelid changing the resistance between the positively charged cornea and the forehead. Accordingly lid fixation severely reduces or even prevents eyelid-related potential changes in the EEG (Chioran and Yee, 1991). Blinks are also accompanied by an active small extorsional, downward, and nasalward eye movement which is followed by a fast passive return to the pre-blinking position due to passive elastic forces (Bour et al., 2000; Bergamin et al., 2002). Yet artifacts arising from such blink-associated eye movements are usually occluded by the lid induced artifact and therefore only play a minor role with respect to blink-related signal contamination.

Importantly, the distribution of eyelid artifacts at scalp level is independent of corneo-retinal dipole orientation. As our results show, their topographic pattern does not change across different types of eye and eyelid movements, and their related potentials only differ in amplitude and duration (see also Evinger et al., 1991; Guitton et al., 1991; Lins et al., 1993b; VanderWerf et al., 2003). Still, eyelid artifacts are interrelated with the corneo-retinal dipole in the sense that an intact eye globe is necessary for the production of blink potentials. Active or passive movements over a non-metallic prosthetic eye do not result in blinking potentials (Matsuo et al., 1975). Conversely, passive eyelid movement over an intact eye results in electrical changes congruent with the ones of blinking and eyelid artifacts (Matsuo et al., 1975).

Another important issue concerning the interrelation between eye and eyelid artifacts is that saccadic eyeball rotations are accompanied by ballistic eyelid movements, or “eyelid-saccades,” that are performed in synchrony with saccadic eye movements (Becker and Fuchs, 1988; Evinger et al., 1991; Guitton et al., 1991; Helmchen and Rambold, 2007). Saccades and eyelid-saccades are coordinated by several brainstem structures and different cortical pre-motor and motor areas (Helmchen and Rambold, 2007). Consequently, eyelid-saccade parameters are closely matched with those of eye-saccades (Becker and Fuchs, 1988) and therefore cannot be disentangled in the signal measured at scalp electrodes. However, after the termination of both eye and eyelid saccades the lid continues to slide for another 30–300 ms (Becker and Fuchs, 1988). This post-saccadic eyelid movement produces a returning change in offset which—in concordance with both, previous studies and our data—is exclusively observed after vertical and oblique upward saccades (Barry and Jones, 1965). However, based on the interpretation of source dipole modeling of eye artifacts, other authors have proposed that post-saccadic eyelid artifacts could also be present for horizontal saccades (Lins et al., 1993a). Although we do not see corresponding changes at scalp level, we cannot discard this possibility, especially as eyelid related ICs display offset changes also during horizontal saccades. Note, that post-saccadic eyelid artifacts are not related to eye movement overshoots as they could be interpreted at first sight (Barry and Jones, 1965). This is supported by the observation that the returning change in offset is not present for eye movements performed with eyes closed (Huddleston, 1970).

Finally, the observation that velocity and duration profiles of post-saccadic eyelid movements and eyelid movements during opening the eyes after blinking are very similar, further argues for the same underlying mechanism (Evinger et al., 1991). However, blinks produce about 5–10 times larger artifacts than post-saccadic eyelid movements. In frontal electrodes, that is about 100 μV for blinks as compared to approximately 20 μV for eyelid artifacts succeeding large upward movements when the recorded data is nose-referenced.

In summary our results confirm that the propagation of eyelid related artifacts onto the scalp is independent of corneo-retinal dipole orientation while their amplitude and latency may change significantly depending on the type of eye and eyelid movement.

#### Spike potential

The saccadic spike potential is a transient high amplitude potential observed around saccade onset. Its origin has been a matter of debate, but although some authors argue for the saccadic spike potential to emerge from cortical sources (Kurtzberg and Vaughan, 1982; Nativ et al., 1990; Parks and Corballis, 2008), it is now generally believed that saccadic spike potentials reflect the recruitment of motor units in extra-ocular muscles (Thickbroom and Mastaglia, 1985, 1986; Picton et al., 2000; Keren et al., 2010; Carl et al., 2011). The onset of the saccadic spike potential precedes the onset of the saccade about 5 ms and therefore this artifact is sometimes referred to as “pre-saccadic” spike potential (Thickbroom and Mastaglia, 1985). However, it has been argued that saccadic spike potentials and saccades actually start at exact the same time but since the latter are usually defined as the eye movement exceeding a certain velocity threshold, their detection lags behind the actual movement onset (Keren et al., 2010).

Both, amplitude and topography of the saccadic spike potential at scalp level change depending on movement size and direction. Yet, the results in the present study suggest that the observed topographic patterns might reflect a mixture of corneo-retinal dipole orientation and different sequences of muscle activation. More specifically we observed that both, amplitude and topography of the saccadic spike potential, are dependent on initial eye position rather than on saccade size. This may indicate that at saccade onset the impact of the corneo-retinal dipole on the overall polarity on the scalp contributes more to the observed topographic pattern than the saccadic spike potential itself.

It appears virtually impossible to study the saccadic spike potential's contribution to the measured signal independently of other artifact sources, because—as it was shown here and in previous studies (Keren et al., 2010)—ICA often fails to single out the saccadic spike potential into one or several separate components. One of the reasons, next to its short duration, may be that different ocular muscle units are recognized as different sources, which however are too weak to be isolated into independent components. Moreover, the problem of studying the properties of the saccadic spike potential during different types of eye movements in isolation of other artifacts will also not be easily resolved by using other methods such as source localization, since their spatial resolution is limited and the precise origin of the mechanisms generating the spike potential is not well understood yet.

#### Microsaccades

Microsaccades are performed involuntarily and their execution is controlled by the superior colliculus (Hafed et al., 2009). Their behavioral purpose is not entirely clear yet but several studies suggest that they play an important role for counteracting visual fading (Martinez-Conde et al., 2004) and enhancing fine spatial detail (Rucci et al., 2007). However, it remains an open question how these putative functions of microsaccades can be brought in accordance with earlier findings that microsaccades are sometimes suppressed in high-acuity tasks like threading a needle or shooting a rifle (Winterson and Collewijn, 1976; Bridgeman and Palca, 1980).

As pointed out above, the most prominent microsaccade-related confounds in EEG data are produced by the spike potential at microsaccade onset, while offsets that go along with corneo-retinal dipole rotation only have a minor impact on the signal.

Consistent with earlier reports (Keren et al., 2010), our results show that ICA-correction significantly reduces the amplitude of microsaccade-related spike potentials. Moreover, the finding that during fixation trials the correction procedure leads to a signal reduction that follows the observed microsaccade distribution suggests that microsaccade-related spike potentials do not only affect the induced gamma band response in the frequency domain but also ERP amplitudes. Thus, addressing the question which factors systematically modulate the rate of microsaccades will not only shed light on the behavioral purpose of microsaccades but may possibly also reveal spike potential induced confounds in previous ERP studies.

In summary, the different types of eye artifacts investigated here are largely independent of each other and display different properties with regard to different types of eye movements: amplitude and topography of corneo-retinal dipole-related artifacts are determined by both, eye movement size and direction. Blink-related artifacts on the other hand are independent of eye movements and although small eye movements do occur during blinking, their impact on the signal is minor as compared to the eyelid induced resistance change. As a consequence the only factor significantly modulating the amplitude of blink artifacts is whether a blink is performed voluntarily or involuntarily. In this respect blink artifacts differ from post-saccadic eyelid artifacts, which do depend on both, eye movement size and direction. More specifically, at scalp level they are only observed during upward eye movements and display larger amplitudes after large saccades. However, apart from differences in amplitude all eyelid-induced artifacts produce the same topographic pattern. Similarly, the topography of the saccadic spike potential changes relatively little for different saccade amplitudes and directions and the observed differences in amplitude seem to depend on the initial eye position rather than on the type of movement. Apart from the saccadic spike potential, the contribution of ocular muscle activity to eye and eyelid movement-related artifacts is negligible (Mowrer et al., 1935). This holds for both, tonic muscle activity that keeps the eye or eyelid in a given position and phasic muscle activity during blinking.

### Correction of eye movement artifacts

#### Regression-based correction

It is widely recognized that blink and saccade-related artifacts propagate differently onto the scalp (Corby and Kopell, 1972; Gratton et al., 1983; Antervo et al., 1985; Elbert et al., 1985; Berg and Scherg, 1991; Picton et al., 2000; Croft et al., 2005). Still, some authors advocate that both types of artifacts can be fully corrected by regression methods that are based on only two orthogonal (i.e., horizontal and vertical) EOG measures, thus ignoring the independence of eyelid and corneo-retinal dipole-related artifacts and the topographic differences between them (Verleger et al., 1982; Schlögl et al., 2007). Other authors that acknowledge these differences argue that in principle artifacts produced by eyelid movement during blinking and upward saccades could be separated from the EOG by including a radial component in the corneo-retinal dipole model (Elbert et al., 1985; Croft and Barry, 2000a,b; Schlögl et al., 2007). However, our results demonstrate that a three-component model does not necessarily result in better correction performance than a two-component model. Therefore it appears doubtful that the effects of eyelid movement on the corneo-retinal dipole can be fully modeled by a single dipole in three dimensions. It has been shown that a correct modeling of both, vertical eye movements and blinks requires at least two current dipoles (Antervo et al., 1985). Also, the best dipole fits for eye movement artifacts are obtained when dipoles are allowed to take different locations and orientations depending on the type of movement. More specifically, dipoles that correspond to blink-related activity should not only be located in front of the vertical eye movement dipole (as it could be modeled by an axial component) but also above it (Berg and Scherg, 1991; Lins et al., 1993a). In addition, it is not clear how to estimate an axial spatial component for a single corneo-retinal dipole source most adequately, as it may depend on electrodes at many different locations (Nunez and Srinivasan, 2006), which are not necessarily symmetric and could contain contributions from neural sources. In the present study we re-referenced channels to mathematically linked temporal channels for calculating the axial component (Elbert et al., 1985; Croft and Barry, 2000a,b). This however resulted in suboptimal correction coefficients for both, blinks and eye movements.

Another problem of regression-based methods is that the regression coefficients are affected by other sources of correlation between EEG channels and EOG channels, principally by brain sources that propagate to both groups of channels and by other electrical artifacts like coherent direct-current shifts (Croft and Barry, 1998a,b). Such correlations could “inflate” or “deflate” regression coefficients in a manner that depends on both, the size of the artifacts and the ratio between confounded and artifact-free periods in the data. For example, regression coefficients calculated with small saccade amplitudes will be inflated with respect to those obtained from larger saccades (Croft and Barry, 1998a,b). To overpass the problem of inflation it has been proposed to subtract event-related activity from the raw data before calculating the coefficients and in this way to eliminate inflation caused by the forward propagation of time-locked neural signals into the EOG channels (Gratton et al., 1983). An alternative proposal suggests that before the calculation of the regression weights, the data should be averaged with respect to artifact events instead of neural events, thereby reducing inflation that is produced by brain activity that is not time-locked to the ocular movement (Croft and Barry, 1998a,b). Although these methods can help to reduce the correlation between EEG and EOG channels, they are not able to completely decorrelate both types of signals. As a consequence inflation of regression coefficients cannot be completely avoided and activity from neural sources may be subtracted from the signal.

To summarize, regression initially appears to provide a straightforward solution for removing eye movement artifacts from the EEG. But as our results show, it is not possible to correct corneo-retinal dipole and eyelid artifacts at the same time. Additionally potential correlations between EEG and regression channels may lead to inflation of regression coefficients and erroneous removal of brain activity from the signal. Note that choosing more advanced regression methods or different regression channels may have yielded better results as the ones presented here. However, the general problems of regression-based eye artifact correction remain and therefore inevitably lead to suboptimal correction performance.

#### Independent component analysis correction

ICA does in principle not suffer from the limitations that are encountered with regression methods. ICA decomposes the signal measured at scalp level into the activity of the particular sources that contribute to the signal. Thus, in the ideal case, brain and artifact-related activity are clearly separated and can be handled independently. In practice however there are several problems related to this approach.

First, the effectiveness of ICA strongly depends on the quality of the signal decomposition. Not all signal sources may be isolated into separate components and there are no definite means to evaluate whether or not contributions of other sources confound a particular component. However, with optimally prepared data (i.e., sufficient length, cleaned from large amplitude noise and rare artifacts and possibly filtered to the frequency range of interest) this problem can be minimized (Makeig and Onton, 2009). A large number of studies has shown that ICA is able to isolate all relevant signal sources in a way that the components of interest, as for example brain activity from a defined area and/or related to a certain cognitive task, are not affected by other components, like artifacts or other cortical sources (Makeig et al., 1996; Makeig and Onton, 2009; Gramann et al., 2010; Gwin et al., 2010).

A second problem is how to objectively identify components related to ocular artifacts. Often this is done based on visual inspection of the components' topography (Jung et al., 2000; Iriarte et al., 2003; Makeig and Onton, 2009), thus relying on the subjective judgment of the experimenter. Here we have shown, that this approach, not only leads to suboptimal detection and possible misclassification of ICs, but also to divergent results depending on the subjective view of the researcher. Likewise, even more objective methods, like defining ocular artifacts based on their statistical properties may fail to identify all relevant components, as different artifacts display very different features with respect to their propagation pattern, amplitude or frequency range (Makeig and Onton, 2009). Another conventional method to identify ocular components is to cluster ICs based on their source locations and defining the signals emerging from clusters located in and around the eye and eyelid as eye movement-related (Gramann et al., 2010; Gwin et al., 2010). However, source clustering might fail in some cases. For example, noisy components often contain eye artifacts but do not display bipolar patterns and thus are likely to be mislocalized.

Here we proposed a procedure to identify eye artifact-related ICs, by comparing their activations during saccade and during fixation intervals, as defined by high temporal resolution eye tracking. In this way we circumvent the problem of how to objectively distinguish artifactual from non-artifactual ICs to a large extent, as this approach does not rely on subjective interpretation of topographies or prior assumptions about artifact properties, such as source location or spectral content. Consequently, the procedure potentially also identifies confounds that are not directly produced by ocular sources but by artifacts that may occur during eye movements in a systematic manner, as for example head or face movement-related muscle artifacts.

We were able to demonstrate that eye movement-related ICs are almost completely separable from other ICs only based on their saccade/fixation variance ratio. The selection algorithm presented here performed more effectively and reliable than human experts, when those had to base their decision on topographies alone or in combination with only a small subset of component activations. The experts outperformed the automated selection procedure when they could assess ICs based on both, their topographies and a large amount of trials. However, given the number of ICs and trials (here: 896 ICs with an average number of 283 trials each) this approach may not be feasible for most experiments. Employing automated IC classification therefore could prove as an efficient alternative, especially since the possibly misclassified ICs in our experiment were most likely related to noise rather than to brain activity.

There are, however, potential drawbacks to the proposed approach: most importantly, brain sources that are mainly active during the saccade interval might be erroneously excluded. But as motor planning and sensory processing usually take place before and after the saccade itself, the exclusion of brain-related components is rather unlikely. In the data presented here, none of the ICs that were identified as eye movement-related appeared to contain contributions from neural sources.

It also has to be noted that the proposed approach, namely classifying ICs according to their activation differences between saccade and fixation intervals, is not entirely free from prior assumptions. As discussed above, small eye movements also occur during fixation and therefore might bias the saccade/fixation variance ratio. However, microsaccades generally occur only in a subset of fixation intervals, and are even less frequent when eye movements are allowed during the experiment. Additionally, spike potentials, which have been identified as the main microsaccade-related confound in EEG data (Yuval-Greenberg et al., 2008; Keren et al., 2010), are not likely to contribute as much to the variance of generally longer fixation periods as to the usually much shorter saccade intervals. Beside these assumptions, the ratio-threshold defining whether a given IC is eye movement-related or not, was set based on heuristics. For our data the pre-defined value of 1.1 proved to be optimally chosen. In other experiments, however, the discrimination threshold may have to be adapted. On the other hand here all ICs with ratios lower than 1.1 but above about 0.9 appeared to be exclusively related to muscle activity and noise, respectively. Therefore we conclude that even setting the threshold to 1 is not likely to affect task relevant neural signals.

Another limitation is that the IC selection procedure we suggested here requires high temporal resolution eye tracking, which may not always be available in standard EEG experiments. It may however be possible to extract saccade periods based on typical eye movement signatures in the EOG channels, such as signal deflections and/or the saccadic spike potential (Keren et al., 2010).

Lastly, effective eye artifact correction using ICA does not only depend on a sufficient amount of eye movements in the data but also on their amplitude. Applying the procedure on another data set containing fewer and smaller saccades resulted in a suboptimal ICA decomposition. Therefore it is highly recommended to include a variety of eye movements that are performed independently from the task at hand in the ICA decomposition, as we did in our pre-experimental procedure.

#### Neural activity accompanying eye movements

Studying brain activity in the presence of eye movements entails another, often overlooked aspect that cannot be solved by artifact correction: the contribution of eye- and eyelid movements to the signal measured on the scalp does not only consist of non-cortical sources. They are also accompanied by cortical activity related to motor preparation, perceptual suppression, and sensory responses. As blinks and eye movements are also present in experimental designs that require fixation, their neural concomitants are easily overlooked especially when relying on artifact correction procedures. Thus, in the presence of voluntary or involuntary eye- and eyelid movements, comparisons between experimental conditions may be influenced by systematic variation of brain activity related to eye movement execution, attentional control (Glimcher, 2003; Müri, 2006; Corbetta et al., 2008), visual suppression and stability during the saccade (Wurtz et al., 2008) or the related visual responses (Dimigen et al., 2009, 2011; Ossandon et al., 2010). Neglecting these potentially systematic neural signals may lead to an erroneous interpretation of the data. Similar to saccades, blinks and eyelid movements are also partially controlled by cortical structures. In monkeys several frontal and parietal areas have been linked to the control of blinks (Gong et al., 2005). In humans, activity in the medial frontal gyrus (Yoon et al., 2005) has been related to spontaneous blinking and activity in precentral motor areas to voluntary blinking or the active inhibition of it (Kato and Miyauchi, 2003; Yoon et al., 2005; Chung et al., 2006). Furthermore blinking has also an impact on sensory processing, as it causes suppression of activity in visual, parietal and prefrontal cortices (Bristow et al., 2005), thereby leading to a decrease of visual sensitivity around the blinking period (Volkmann et al., 1980). Additionally the interruption of visual input while the eyelid covers the pupil, a period that may range from 100 to 300 ms, usually goes unnoticed. This phenomenon, commonly termed as “visual continuity,” has been related to activity in parieto-occipital areas during blinking (Bristow et al., 2005). Although not consciously processed, blink-related visual potentials do display differences depending on the degree of illumination (Berg and Davies, 1988).

Lastly, due to the occurrence of microsaccades, eye movement-related brain signals are likely to be present even during fixation intervals. Dimigen et al. (2009) have recently shown that microsaccades are not only accompanied by extra-cortical confounds (i.e., spike potentials) but also by activity emerging from cortical sources. They found that microsaccades generate visually evoked potentials, so called lambda responses, as they typically occur in response to stimulus onset or after larger voluntary saccades (Dimigen et al., 2009).

Altogether these findings imply that eye movements influence the recorded EEG in a way that cannot be separated from neuronal processing. Therefore experimenters should be aware that frequency and size of eye and eyelid movements may vary systematically between conditions: saccade rate for instance depends on a variety of aspects like attention, task and image features. Also, the probability of fixational eye movements is known to change in dependency of behavioral task (Winterson and Collewijn, 1976; Bridgeman and Palca, 1980), proportion of target stimuli in oddball paradigms (Dimigen et al., 2009) and image type (Rucci et al., 2007; Yuval-Greenberg et al., 2008). Similarly, size, frequency, and timing of blinks were found to depend on different cognitive and experimental factors such as attentional breaks (Siegle et al., 2008; Nakano and Kitazawa, 2010), mind-wandering (Smilek et al., 2010), use of startle stimuli (Lang et al., 1990), and the occurrence of certain saccade types as for instance while changing lines during reading (Orchard and Stern, 1991).

In summary, relying on artifact correction methods alone would mean to ignore the fact that eye movements do not only introduce artifacts to the EEG but that they also go along with neural activity, which when overlooked may lead to misinterpretation of the data. But eye movements are an essential part of human cognition and experimental setups where they have to be suppressed may not provide adequate information about neural processing under natural conditions. Moreover, systematic variations of eye movements between conditions directly result from differences in cognitive processing and therefore their respective neural signatures cannot be dismissed as “confounds”. In many cases, especially when studying overt visual attention, these alleged “neural confounds” constitute exactly the activity of interest. Thus, eye movements during EEG recordings should not necessarily be considered as interferences but as a part of natural human behavior. Their presence, however, demands the experimenter's awareness and the observed patterns of neural activity have to be interpreted carefully in the sense that they may be linked to visual attention and saccade execution rather than to other cognitive processes that may be the actual focus of interest. Under this point of view simultaneous eye tracking or other saccade detection methods, may help to identify possible biases that arise from systematic differences in probability, size, and timing of eye- and eyelid movements (cf. Kierkels et al., 2007; Yuval-Greenberg et al., 2008; Keren et al., 2010; Dimigen et al., 2011).

## Conclusion

A large number of studies have investigated individual eye movement artifacts under various aspects (e.g., Thickbroom and Mastaglia, 1985; Chioran and Yee, 1991; Lins et al., 1993a,b; Helmchen and Rambold, 2007; Keren et al., 2010) and a variety of different methods has been proposed to correct or reduce their impact on the EEG (e.g., Lins et al., 1993a; Croft and Barry, 2000a,b; Jung et al., 2000; Iriarte et al., 2003; Schlögl et al., 2007). There are efforts to optimize these methods in order to overcome some of their inherent limitations (e.g., Gratton et al., 1983; Croft and Barry, 1998a,b; Makeig and Onton, 2009; Keren et al., 2010) and it has been suggested to complement EEG measurements with eye-tracker information in order to address the problems and pitfalls that are connected to recording EEG in the presence of eye movements (e.g., Kierkels et al., 2007; Dimigen et al., 2011).

Here, by co-registering EEG and eye movements, we studied a wide range of eye artifacts and reinvestigated a number of previous findings within one single data set. This made it possible to examine eye artifacts not only with respect to their individual properties but also their interrelations, thus making earlier findings more comparable. In addition we assessed the efficiency of regression and ICA based artifact correction methods. Unlike earlier studies we also evaluated their impact on a well-defined visual brain response, which is suited to serve as a reference for direct statistical comparisons in both the time and frequency domain. Finally we propose a procedure for the automated selection of eye movement-related ICs. As the procedure is based on a single quantifiable criterion (i.e., the variance ratio of their activations) it can be equally applied to all types of eye movement-related artifacts, without requiring individual decisions by the experimenter.

### Conflict of interest statement

The authors declare that the research was conducted in the absence of any commercial or financial relationships that could be construed as a potential conflict of interest.
